# Human milk oligosaccharide composition and associations with growth: results from an observational study in the US

**DOI:** 10.3389/fnut.2023.1239349

**Published:** 2023-10-03

**Authors:** Fabio Mainardi, Aristea Binia, Purva Rajhans, Sean Austin, Sean Deoni, Nora Schneider

**Affiliations:** ^1^Nestlé Institute of Health Sciences, Nestlé Research, Société des Produits Nestlé S.A., Lausanne, Switzerland; ^2^Nestlé Institute of Food Safety and Analytical Sciences, Nestlé Research, Société des Produits Nestlé S.A., Lausanne, Switzerland; ^3^Advanced Baby Imaging Lab, Rhode Island Hospital, Warren Alpert Medical School at Brown University, Providence, RI, United States; ^4^Department of Radiology, Warren Alpert Medical School at Brown University, Providence, RI, United States; ^5^Bill & Melinda Gates Foundation, Seattle, WA, United States

**Keywords:** human milk oligosaccharides, child development, longitudinal modeling, cluster analyses, statistical methodologies, infant nutrition (including breastfeeding)

## Abstract

**Background:**

Breast milk is the recommended source of nutrients for newborns and infants. Human milk oligosaccharides (HMO) are the third most abundant solid component in human milk and their composition varies during lactation.

**Objectives:**

Our objective was to investigate longitudinal and cross-sectional changes in HMO composition and whether these changes were associated with infant growth up to 24 months of age. Associations with maternal characteristics were also investigated.

**Methods:**

24 HMOs were quantified in samples taken at 2 weeks (*n* = 107), 6 weeks (*n* = 97) and 3 months (*n* = 76), using high performance liquid chromatography. Body length, weight, and head circumference were measured at 8 timepoints, until 24 months. Clusters of breast milk samples, reflecting different HMO profiles, were found through a data-driven approach. Longitudinal associations were investigated using functional principal component analysis (FPCA) and used to characterize patterns in the growth trajectories.

**Results:**

Four clusters of samples with similar HMO composition were derived. Two patterns of growth were identified for length, body weight and head circumference via the FPCA approach, explaining more than 90% of the variance. The first pattern measured general growth while the second corresponded to an initial reduced velocity followed by an increased velocity (“higher velocity”). Higher velocity for weight and height was significantly associated with negative Lewis status. Concentrations of 3’GL, 3FL, 6’GL, DSNLT, LNFP-II, LNFP-III, LNT, LSTb were negatively associated with higher velocity for length.

**Conclusion:**

We introduced novel statistical approaches to establish longitudinal associations between HMOs evolution and growth. Based on our approach we propose that HMOs may act synergistically on children growth. A possible causal relationship should be further tested in pre-clinical and clinical setting.

## Introduction

Human milk oligosaccharides (HMO) are the most abundant solid component of human milk, after fat and lactose, with an estimated concentration ranging between 5 and 15 g/L (1). Although largely not digestible by the infant’s gut, HMOs are known to be associated with a range of biological functions, such as gastrointestinal development (2–8), protection against infection (9–11) and inflammation (12) and more recently in neurodevelopment (13). Breastmilk composition, and in particular the concentration of HMOs, is known to evolve during the lactation period (1, 14, 15); the total concentration of HMOs has been observed to decrease, as well as the concentration of most of the individual HMOs, with the notable exception of 3FL which increases over time (1, 15, 16). Berger et al. (17) have speculated that these dynamic changes in HMO concentrations may reflect their involvement in biological functions beyond the first few months.

The concentration of HMOs in breast milk varies among individuals and particularly depends on the expression of α1-2-fucosyltransferase (FUT2) and α1-3,4-fucosyltransferase (FUT3) genes which determine the secretor (Se) and Lewis (Le) status, respectively (18). 2′-fucosyllactose (2’FL) is absent in the milk of non-secretor women, while it is the most abundant HMO in the milk of secretors. While maternal genetic polymorphisms for the Se and Le genes explain, to a large extent, the HMO variability between mothers (18), additional maternal or infant factors like pre-pregnancy BMI, age, mode of delivery and gestational age may contribute as well to the interindividual variability (15).

The first objective of this exploratory work was to characterize the temporal and inter-individual variability of the HMO concentrations in a US cohort over the first 3 months of lactation. 24 HMOs were measured at three time points; although approximately 150 HMOs have been identified in the literature, it is known that the HMOs used in our study account for roughly 90% of the total HMO (1). We developed a novel data-driven approach to cluster breast milk samples based on the concentrations of HMOs, and tested if these clusters were associated to maternal factors, such as mode of delivery or pre-pregnancy BMI. The data-driven clustering was based on the abundances of all the measured HMOs, assigning samples to milk types sharing similar HMO profiles, and we compared the resulting clusters to the milk types defined by the presence/absence of specific HMOs (e.g., 2’FL and LNFP-I) as proxies for the Se, Le status (18). Van Leuween (19) observed that it may not always be correct to determine the milk type based on HMOs composition at one single time-point, and in our analysis we also considered the intra-individual variability, by describing trajectories of individual HMOs within each cluster.

As a second objective, we investigated the effect of HMO concentrations on growth parameters (head circumference, body weight and body length, measured at 8 time points up to 24 months of age). HMOs are hypothesized to affect growth through several mechanisms, including a possible role in functional gut maturation, therefore improving nutrient absorption (20). Moreover, in some studies, multiple HMOs (eg 2’FL, and 3FL) have been detected in the urine and plasma of breastfed infants, but not in formula-fed infants (21), suggesting the possible existence of systemic effects, yet to be identified (14, 16, 22–27).

The biological functions of many, if not most, HMOs remain unknown. Selected HMOs are increasingly added to infant formulas for their beneficial health benefits (6). It is therefore important to understand better these benefits, whether they might be supported by synergistic effects and what are the natural variations of the individual HMO concentrations over the course of lactation. This knowledge might help to target the HMOs that are the most relevant at critical windows of infant development. In turn, such investigations pose methodological challenges for the data analysis, that the present study intended to address.

Patterns of development are often non-linear, therefore linear mixed effect models that are often used in modelling growth trajectories may not describe the data accurately. Non-linear parametric approaches are an alternative to linear mixed models, however, they rely on parametric assumptions that might not be realistic. In addition, the sparsity often found in real data is a challenge for fitting the data into those models (23). Functional data analysis offers a non-parametric, non-linear approach to model sparse, non-linear longitudinal data. The FPCA approach allows extracting the patterns of growth from a set of trajectories and has been successfully applied in many domains, including neurodevelopment (28) and gene expression (29, 15).

## Materials and methods

### Endpoints

Table 1 summarizes the timepoints available for the milk samples and the body measures.

**Table 1:** Overview of time points.

	V0 (2–5 week)	V1 (6 ± 1 week)	V2 (3 month ± 2 week)	V3 (6 month ± 2 week)	V4 (9 month ± 2 week)	V5 (12 month ± 2 week)	V6 (18 months ± 3 week)	V7 (24 month ± 4 week)
Breast milk samples	X	X	X					
Body measures	X	X	X	X	X	X	X	X

Body measures include head circumference body length and body weight.

### Description of study population

Children for this study were selected from the observational breastfeeding arm of a prospective longitudinal randomized control trial. Results for the randomized arm were analyzed in a previous publication (30). Infants for the trial were recruited and followed over 24 months at two study sites in the United States (Rhode Island Hospital in Providence, RI, and Pennington Biomedical Research Center, in Baton Rouge, LA). Maternal screenings were performed during the third trimester of pregnancy up to and including post-delivery. Following written informed consent (screening visit), sociodemographic information, medical and family histories were collected, as well as a physical and neurological examination of the infant. Withdrawal from the study was possible at any point and with no further evaluations and any additional data collection. The research ethic boards at both clinical sites approved the protocol.

In total, 107 breast milk samples were available for analysis. The demographic characteristics of the mothers are summarized in Table 2. 61 (57%) children were female, 46 (43%) were male. Mean age of the mothers at recruitment ranged from 19 to 43 years, with an average of 32 years. Most mothers were white (70%) BMI before pregnancy ranged from 19.1 to 39.9, with an average of 27.2 and a median of 26. 72% of children were born through vaginal delivery.

**Table 2:** Maternal characteristics.

Ethnicity	*n*	%
White	75	70
African American, black	8	7
Mixed race	8	7
Hispanic	5	5
Asian	3	3
Latino	2	2
Other	6	5
*Income*	*n*	*%*
I prefer not to answer	23	21
200,000 USD or more	7	6
150,000–199,999 USD	10	9
110,000–149,999 USD	17	16
90,000–109,999 USD	9	8
70,000–89,999 USD	10	9
50,000–69,999 USD	10	9
30,000–49,999 USD	10	9
10,000–29,999 USD	9	8
Missing	2	2
*Number of siblings*	*n*	*%*
0	39	36
1	44	41
2	14	13
3	7	7
4	1	1
5	2	2
*Body measures*	*Mean (SD)*	*[Min, Max]*
BMI before pregnancy	27.3 (5.8)	[19.1, 39.9]
*Gestational age*	*Mean (SD)*	*[Min, Max]*
Weeks	39.3 (1.11)	[37.0, 41.0]
*Age at recruitment*	*Mean (SD)*	*[Min, Max]*
Years	32 (5)	[19, 43]
*Mode of delivery*	*n*	*%*
Vaginal	77	72
C-section	30	28
*Maternal education*	*n*	*%*
High school not graduated (10th or 11th grade)	2	2
High school graduate	8	7
Partial college/university, not graduated	24	22
Profession training/graduate degree/master/doctorate/MBA	30	28
Other	43	40

Supplementary Table 1 summarizes the anthropometric characteristics of the breastfed children involved in the study.

Exclusion criteria were: (i) Birth>41 weeks +6 days gestation as reported in medical record when available; (ii) Birth Weight < 2000 g or small for gestation age (birth weight less than the 10th percentile for the gestational age) or large for gestational age (weight, length, or head circumference above the 90th percentile); (iii) Any unsafe psychopharmacological treatment of mother using prohibited medications during pregnancy or lactation as assessed by medical interview. This included anticonvulsants, antidepressants, benzodiazepines, cytotoxic drugs, dopamine agonists, opioids.

### Milk samples

Breast milk was sampled from mothers longitudinally at defined study visits, each time between 10 AM–12 PM from the right breast using a hospital grade electric breast pump. Mothers were asked to empty the right breast approximately 2 h prior to milk sampling and the time of milk sampling the complete breast was emptied using a pump (single full breast milk sampling methodology). Only a fraction of the collected milk was aliquoted for research and the rest was returned to mother to feed the baby at a later time.

Data on feeding mode and introduction of solid food were collected via questionnaires at baseline, 6 weeks, 3, 6, 9, and 12 months. Up to at least 4 months of age children received breastmilk for more than 90% of nutritional intake and no infant formula or solids for more than 10% of the nutritional intake (approximately 6 feeds per week).

HMO data (Table 3) were available for *N* = 107, 97, 76 individuals at 2–5 weeks, 6 weeks, 3 months (visits V0–V2), respectively. 76 mothers had complete HMO data for all three visits.

**Table 3:** Overview of all HMOs measured, with chemical structure and abbreviation.

HMO		Abbreviation
2'-FUCOSYLLACTOSE	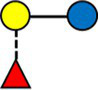	2'FL
3'-GALACTOSYLLACTOSE	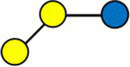	3'GL
3'-SIALYLLACTOSE	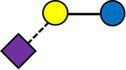	3'SL
3,2'-DIFUCOSYLLACTOSE	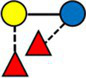	DFL
3-FUCOSYLLACTOSE	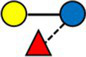	3FL
6'-GALACTOSYLLACTOSE	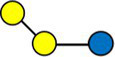	6'GL
6'-SIALYLLACTOSE	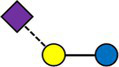	6'SL
A-TETRASACCHARIDE	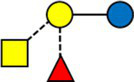	A-TETRA
DIFUCOSYLLACTO-N-HEXAOSE-a	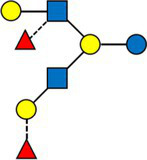	DFLNHa
DISIALYLLACTO-N-TETRAOSE	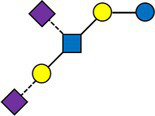	DSLNT
Unidentified hexasaccharide of composition Hex4 HexNAc2	unidentified	Hex4 HexNAc2
LACTO-N-DIFUCOHEXAOSE-I	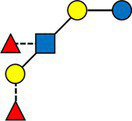	LNDFH-I
LACTO-N-FUCOPENTAOSE-I	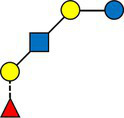	LNFP-I
LACTO-N-FUCOPENTAOSE-II	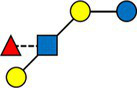	LNFP-II
LACTO-N-FUCOPENTAOSE-III	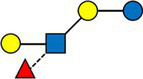	LNFP-III
LACTO-N-FUCOPENTAOSE-V	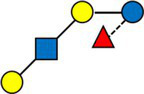	LNFP-V
LACTO-N-HEXAOSE	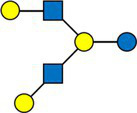	LNH
LACTO-N-NEODIFUCOHEXAOSE	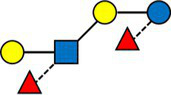	LNnDFH
LACTO-N-NEOFUCOPENTAOSE-V	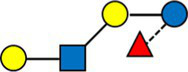	LNnFP-V
LACTO-N-NEOTETRAOSE	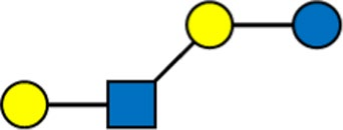	LNnT
LACTO-N-TETRAOSE	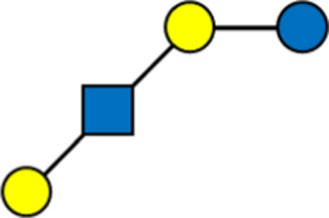	LNT
MONOFUCOSYLLACTO-N-HEXAOSE-III	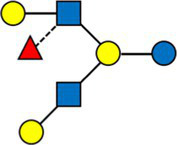	MFLNH-III
SIALYLLACTO-N-TETRAOSE-B	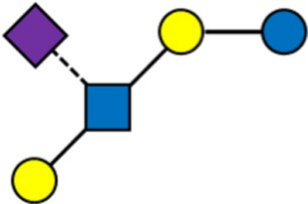	LSTb
SIALYLLACTO-N-TETRAOSE-C	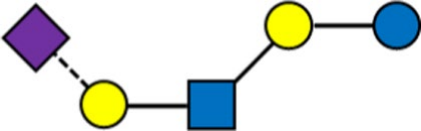	LSTc
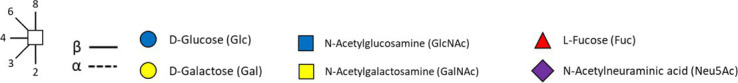		

HMOs were analyzed by ultra-high performance liquid chromatography with fluorescence detection (UHPLC-FLD), according to the method of Austin and Benet (31). We refer to Table 3 for the full list of measured HMOs and their respective abbreviations. 2’FL, 3FL, 3’SL, 6’SL, LNT, LNnT and LNFP-I were quantified against standards of analytical quality all other HMOs were quantified against maltotriose assuming equimolar response factors. Mother’s genotypes to define Secretor and Lewis status were not available.

### Statistical analysis

We first calculated the sum of all HMO concentrations at each time point in each sample and tested for significant differences between visits V0 and V1, and between V1 and V2. We compared the Shannon diversity of the HMOs between timepoints. The Shannon diversity index is low when all HMOs are present in similar concentrations. The more unequal the abundance of HMOs, the larger the corresponding Shannon entropy.

In this work, we analyzed both the proportion of each HMO, expressed as % of the sum of the 24 measured HMOs, and their concentration expressed in mg/L. Since the volume of milk per feeding was not measured, total amounts of HMOs or other milk components are not known, and only relative information was available for analysis.

We applied a data-driven method to assign mothers to clusters of HMO composition. The number of clusters was data-driven as well. Basically, we clustered together samples sharing a similar HMO profile. Our approach was based on network theory (32), where each node of the network corresponds to a milk sample and two nodes are connected based on the similarity between their HMO profiles. We first calculated the Aitchison (33) distances between all the pairs of samples, all subjects and all timepoints. The resulting matrix of distances allows us to identify the closest neighbor to a given sample. The edges in the network were weighted, with weights inversely proportional to the distance between the samples. Therefore, two samples with similar HMO composition have a short distance and therefore will be connected with a high weight in the network. We applied the leading eigenvalues method (34) to identify clusters in the network. These clusters correspond to groups of samples sharing a similar HMO composition. We applied a Kruskal-Wallis test to each HMO to test whether its concentration was significantly different between the clusters. We also made qualitative comparisons with clusters of HMOs that can be derived from presence or absence of 2’FL and LNFP-II (3, 6).

We tested for differences between HMO clusters for several covariates: mother’s BMI before pregnancy, BMI status, mode of delivery, ethnicity, and gestational age. BMI status was defined as ‘overweight’ (OW) for a BMI ≥25 kg/m (2), ‘obese’ (OB) if BMI ≥30 kg/m^2^, ‘normal weight’ (NW) otherwise. Fisher’s exact tests were used to test the independency between two categorical variables. Permutational analysis of variance (PERMANOVA) was used to test the equality of the overall HMO composition between groups (e.g., clusters). Kruskal-Wallis test was used to check the independence between a continuous and a categorical variable, followed by a Dunn *post-hoc* test.

We calculated all pairwise correlations between the individual HMOs, at each timepoint, and visualized them as a heatmap, to summarize and display patterns in the data. In particular, the existence of strong correlations between pairs of HMOs is an important background information when interpreting associations arising from the application of univariate models, and suggests a possible synergistic effect of several HMOs acting in combination.

All correlations reported in this study were Spearman correlations, and a Benjamini-Hochsberg correction for multiple testing was applied whenever applicable, using a false discovery rate of 5%.

Previous work (14, 16) has explored the influence of individual HMOs on growth, using linear mixed models with growth parameters as response variable and the HMO level as predictor. We propose an alternative approach derived from functional data analysis (22–27) to describe the growth trajectories and their main modes of variation; this approach allowed us to identify distinct growth patterns in the data, that could not be properly detected using linear models. These patterns were then investigated in association with the clusters derived in the first part of the analysis.

We investigated the associations between the HMO composition and the child’s growth over time.

We plotted the growth data separately for boys and girls, using the WHO reference values.^1^ Growth trajectories were analyzed within the methodological framework of functional data analysis (23). More specifically, we applied Functional Principal Component Analysis (FPCA) to describe trajectories using a limited number of numerical parameters, the FPCA scores (11). FPCA models longitudinal data as samples from smooth curves, so that the time-varying trait of the *i*-th subject admits a Karhunen-Loeve expansion


$$X_{i}\left(t\right)=\mu \left(t\right)+\underset{k\geq 1}{\sum }\xi_{i,k}\phi_{k}\left(t\right)$$

where $\mu \left(t\right)$ represents the average trajectory and the $\xi_{i,k}$ are the FPCA scores. The sum in the above formula can be truncated to a fixed number of terms, and the fraction of variance explained will depend on how many terms are kept in the sum. We applied FPCA separately to the length, weight, and head circumference data.

For our applications, based on the percentage of variation explained, we selected the first 2 scores as descriptors for the trajectories, FPCA1 and FPCA2. We therefore effectively achieve a dimensionality reduction, where a trajectory, *a priori* defined by 8 time points, can be effectively described by just 2 parameters.

We then tested whether the FPCA scores were associated with the HMO clusters, with the single HMO concentrations or with the maternal baseline characteristics.

We then further investigated which of the HMOs concentrations were associated with higher velocity, by running linear models adjusted for clustering and with an interaction term between cluster and HMO concentration:


(1) FPCA2=Intercept+HMO+cluster+cluster∗HMO

where HMO stands for the HMO concentration in mg/L, cluster is a categorical variable 1–4 and the last term accounts for the interaction.

It is expected that the body length, weight, and head circumference are correlated, therefore we report the correlations between the FPCA scores associated to the various growth measures (length, weight, head circumference).

All analyses were performed with R, version 4.0.2. Network analysis was performed with the package *igraph (v 1.3.0)* and functional PCA was performed using the package *fdapace (v 0.5.8).*

## Results

### Description of HMO composition over the lactation period/time

The total measured HMO concentration decreased over the first 3 months of lactation from 9182 (2013) mg/L, at V0, to 7,887 mg/L (1813) at V1 and 6,248 mg/L (1322) (mean (SD)) at V2.

Consistently with previous findings (15) we observed a decrease of almost all HMOs with time of lactation (Supplementary Table 2). A noticeable exception to this decreasing pattern was 3FL, which increased from 704 mg/L (538 mg/L) at V0 to 1,118 mg/L (698 mg/L) at V2.

Mothers can be deemed as secretors/non-secretors based on the presence/absence of α-1-2-linked fucosylated HMOs (2’FL and LNFPI). In secretor mothers, 2’FL was the most abundant HMO, representing up to 58% of the total measured HMO (Supplementary Table 2).

The Shannon diversity at V2 was lower than at V1 (Wilcoxon test, *p* < 0.01), and lower than at V0 (Wilcoxon test, *p* < 0.01). This is reflected in the fact that the cumulative proportion of the two most abundant HMOs, 2’FL and 3FL, increased from 29% at V0 to 34% at V1 and increased to 41% at V2. There was no significant difference in diversity between V0 and V1.

Pairwise correlations between the concentrations of individual HMOs are reported in the supplementary materials (Supplementary Figures 1–3): significant correlations ranged between −0.9 (2’FL and LNFP-V at V0) and 0.9 (3FL and LNFP-II at V1). In general, we observed very strong positive correlations between 2’FL, DFNLHa, LNFP-I, between 3FL and LNFP-II, and between DSLNT, LSTb, LNFP-V, LNT at all timepoints. Also, Hex4 HexNAc2 was positively correlated (Spearman *ρ* = 0.7 at V0) with LNnT, and 6’SL with LSTc (*ρ* = 0.7 at V0, V1, *ρ* = 0.8 at V2). 2’FL and LNFP-II were not significantly correlated with LNH, 3’GL, 6’GL, 6’SL, LSTc. LNFP-I was significantly and negatively correlated with 3FL (*ρ* = −0.8 at V0), LNFP-V (*ρ* = −0.5 at V0), LNFP-II (*ρ* = −0.5 at V0), LNnFP-V (*ρ* = −0.4 at V0), LNFP-III (*ρ* = −0.3 at V0). In most cases, these correlations and their statistical significance were similar across visits. Other HMOs, like A-Tetrasaccharide or 6’GL, were weakly correlated, or not significantly correlated with the other HMOs.

**Figure 1:**
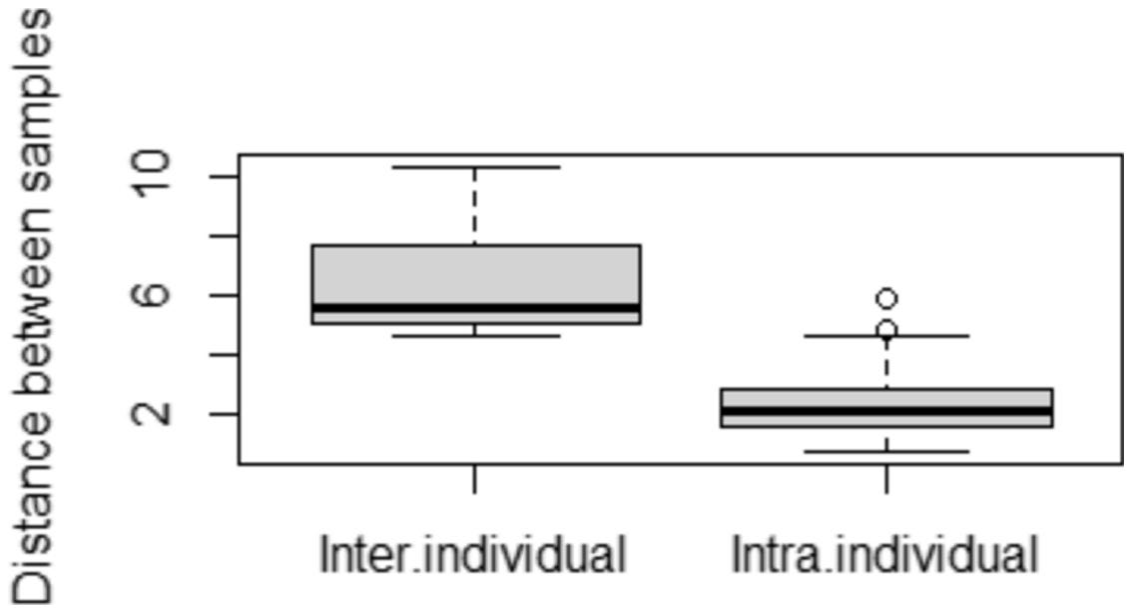
Comparison of distances between samples. Inter-individual distances were calculated between different mothers, and intra-individual distances between samples from the same mother but at different time points.

We calculated the pairwise Aitchison distances between all samples, resulting in a 276 × 276 matrix. Figure 1 compares the distances between samples from the same donors and samples from different donors. As expected, the intra-individual distances between samples were significantly smaller than the inter-individual distances.

We then defined a weighted network with the samples as nodes and edges weighted by the inverse of their distance. Each node corresponds to a subject at a given timepoint. The resulting weighted network was partitioned, producing four clusters of sizes 97(35%), 89 (32%), 62 (22%), 28 (10%), consisting of samples sharing a similar HMO composition.

**Figure 2:**
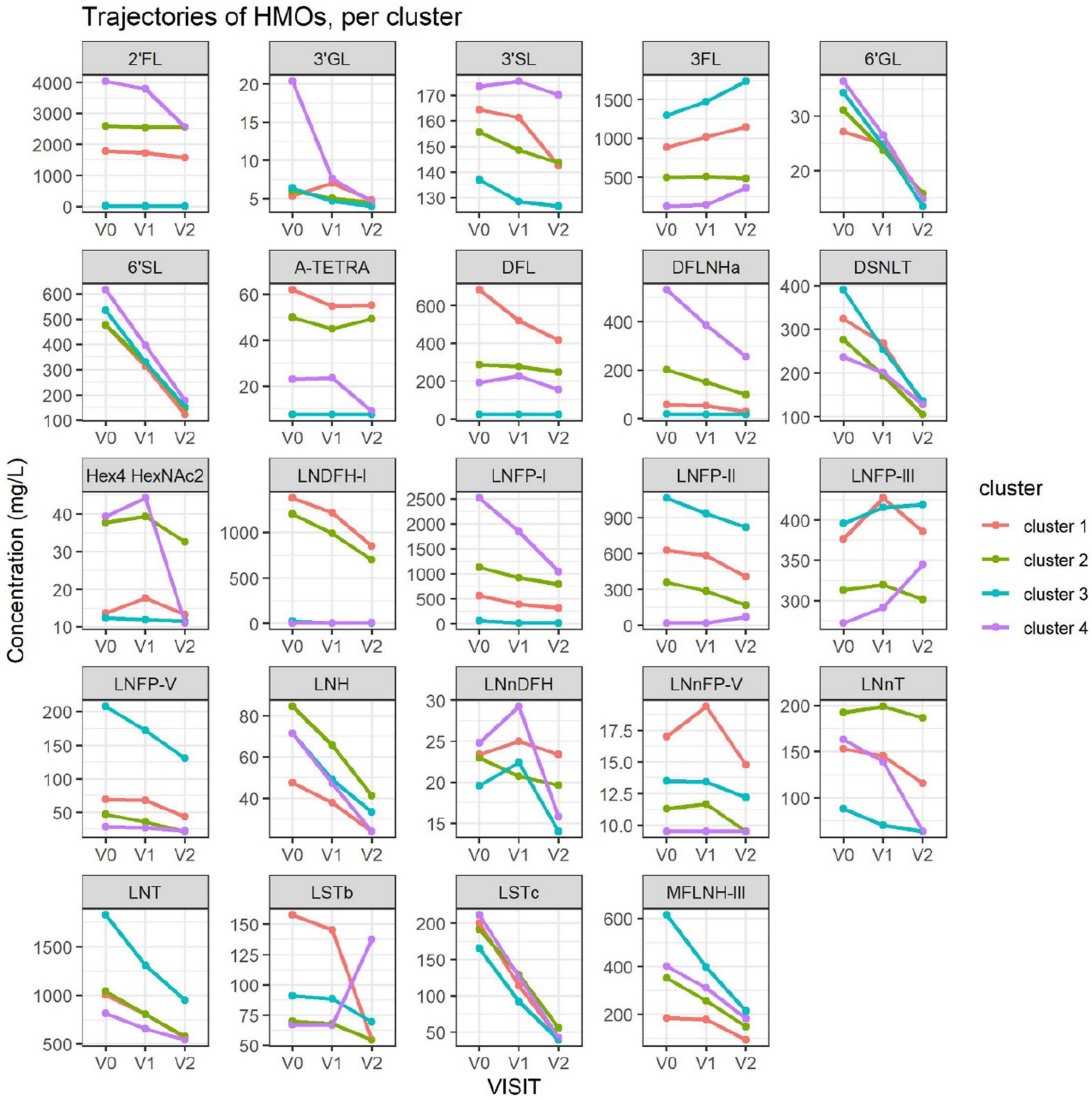
Each dot represents the average concentration of each HMO, in each cluster and for each time point. Several clustering variables show consistent temporal patterns: for example, LNFP-V is consistently highest in cluster 4, while 3FL is consistently highest in cluster 3 (non-secretors). V0 = 2–5 weeks, V1 = 6 weeks, V2 = 3 months. For other HMOs, like 6’GL, LSTc and DSLNT, the concentration is similar between the clusters. Several HMO concentrations exhibit a non-linear trend, with an increase between the first and second visit and a decrease between the second and third visit.

**Figure 3:**
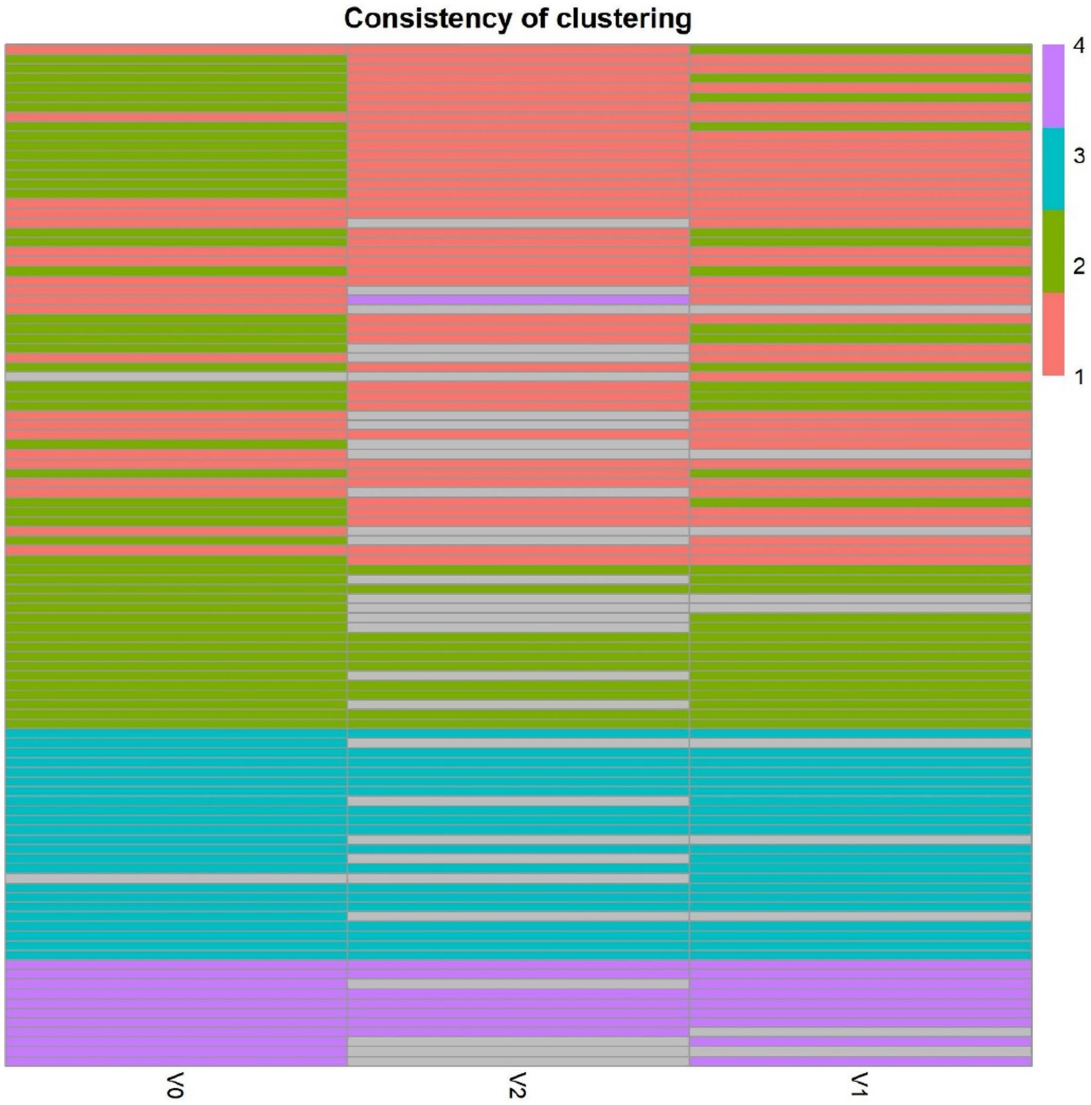
Temporal consistency of clustering: each row corresponds to a mother, the heatmap describes whether the milk samples remained in the same cluster between visits. Grey cells are missing values. All subjects belonging to cluster, 3 or 4 at the first visit, remain in the same cluster afterwards. Some of the subjects starting in cluster 2 (green) switch to cluster 1 (orange) afterwards.

The average value of each HMO at each timepoint and in each cluster was plotted, as concentrations in mg/L (Figure 2). Overall, it appeared that several HMOs (e.g., 3FL, 2’FL, LNFP-I, LNFP-II) had well separated, non-overlapping, trajectories in the different clusters, while for other HMOs (e.g., 6’SL, LSTc, LSTb) the trajectories were overlapping or even crossing. The concentrations of 6’SL decreased in all clusters, from an average of 468 mg/L at V0 to 119 mg/L at V2 in cluster 1, from 489 mg/L to 147 mg/L in cluster 2, from 535 mg/L to 155 mg/L in cluster 3 and from 588 mg/L to 203 mg/L in cluster 4. In Figure 3, we looked at the temporal consistency of clustering: whether the milk samples remained in the same cluster across visits. All subjects belonging to cluster, 3 or 4 at the first visit, remain in the same cluster afterwards. Some of the subjects starting in cluster 2 switched to cluster 1 afterwards.

Cluster 1 was characterized as having the highest concentrations of A-TETRA, DFL, LNDFH-I and LNnFP-V and second highest proportions of 3FL, LNFP-II (Figure 2). It also had the lowest levels of MFLNH-III. Cluster 2 was characterized by the highest concentrations of LNnT and LNH, and second highest concentrations of 2’FL, A-TETRA and LNDFH-I. Also, the concentration of 3FL in this cluster was roughly stable across visits. Cluster 3 was characterized by highest concentrations of 3FL, LNFP-II, LNFP-V, LNT, MFLN-III, and lowest concentrations of 2’FL, LNDFH-I, LNFP-I, LNnT. 2’FL was actually absent in this group, which then corresponds to the non-secretor group. LNFP-I was also absent in this cluster. Cluster 4 is characterized by highest concentrations of 2’FL, DFLNHa, LNFP-I, and lowest concentrations of 3FL, LNDF-I, LNFP-II, LNFP-V. Moreover, LNDFH-I and LNFP-II were absent in this cluster. Clusters 3 and 4 had significantly smaller Shannon diversity at all time points compared to clusters 1 and 2, cluster 4 had a significantly lower diversity than cluster 2 at visits V0 and V1, and cluster 2 had lower diversity than cluster 1 at visit V1.

**Figure 4:**
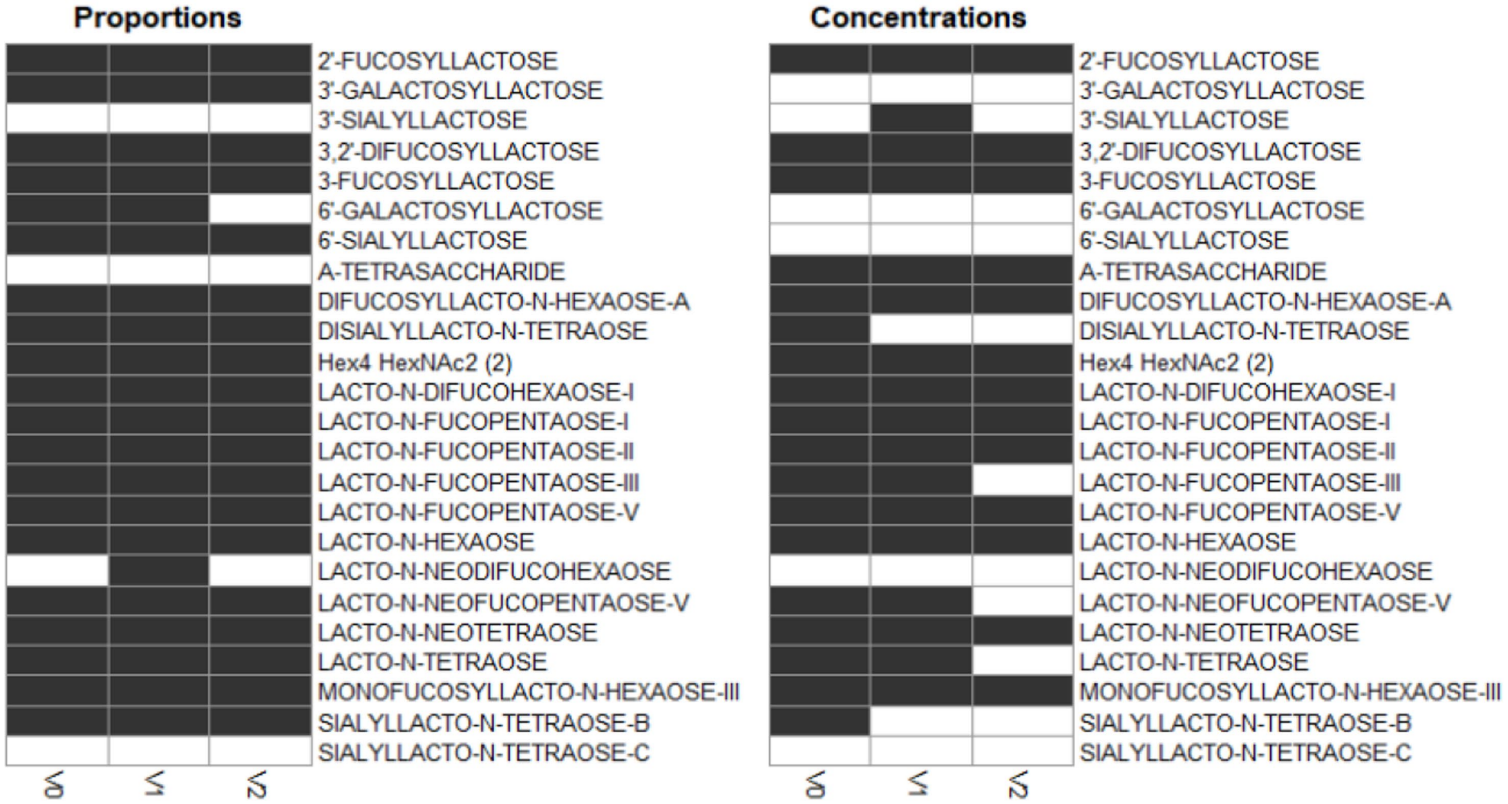
Dark cells correspond to significant difference between the clusters (kruskal test). On the left, HMOs are expressed in relative concentrations (as % of total HMO), on the right, they are expressed in mg/L.

A majority of HMOs had significantly different concentrations between the clusters, at least one timepoint, with the exception of 3’GL, 6’GL, 6’SL, LNnDFH, LSTc (Figure 4).

**Figure 5:**
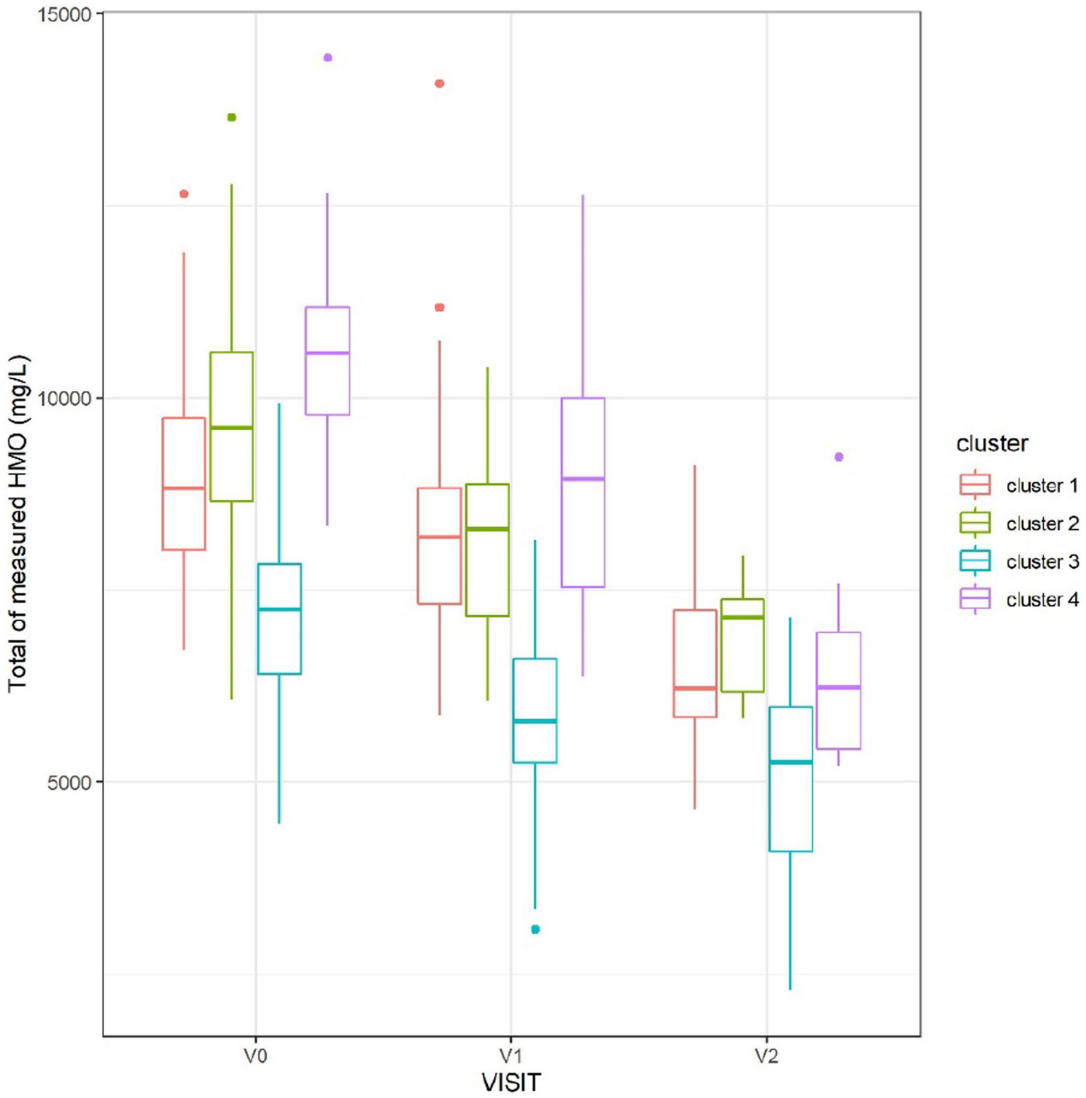
Sum of measured HMO concentrations, at each visit, split by cluster. Cluster 3, corresponding to non-secretors, had the lowest total concentration of HMOs.

The total measured HMO concentration varied between the clusters and was consistently the lowest in cluster 3 (Figure 5).

### Association with maternal and baseline characteristics

HMO clustering was not associated with mother’s age at recruitment, pre-pregnancy body mass index (ppBMI) or gestational age.

We further tested the impact of BMI status on HMO concentrations, First, total HMO concentration and Shannon diversity index were not different between overweight (OW), obese (OB) and normal weight (NW) groups, at any visit. We then compared the average trajectories for each of the HMOs between the BMI groups regardless of clusters, using functional ANOVA (23): tests were significant only for 6’GL (*p* = 0.04), LNH (p = 0.04), LSTc (*p* = 0.04) (Supplementary Figure 4). We also tested if HMO concentrations were different between vaginal delivery and C-section. Specifically, the concentration of LNnT and Hex4 HexNAc2 (2) at 3 months was affected by birth mode and lower in the group of mothers who delivered through C-section (Table 4).

**Table 4:** Concentrations of Hex4 HexNAc2 and LNnT at 3 months are lower in the C-section group.

HMO	C-section Mean concentration (mg/L)	Vaginal delivery Mean concentration (mg/L)	Adjusted *p* value
Hex4 HexNAc2	11.2	16.7	0.048
LNnT	85.6	116.0	0.039

### Association with growth

Children in this study showed a normal development for length and weight (see Supplementary Figures 5–8).

Although the differences did not reach statistical significance, children from mothers in cluster 3 (non-secretors) were on average 1 cm shorter than the rest.

#### Length

**Figure 6:**
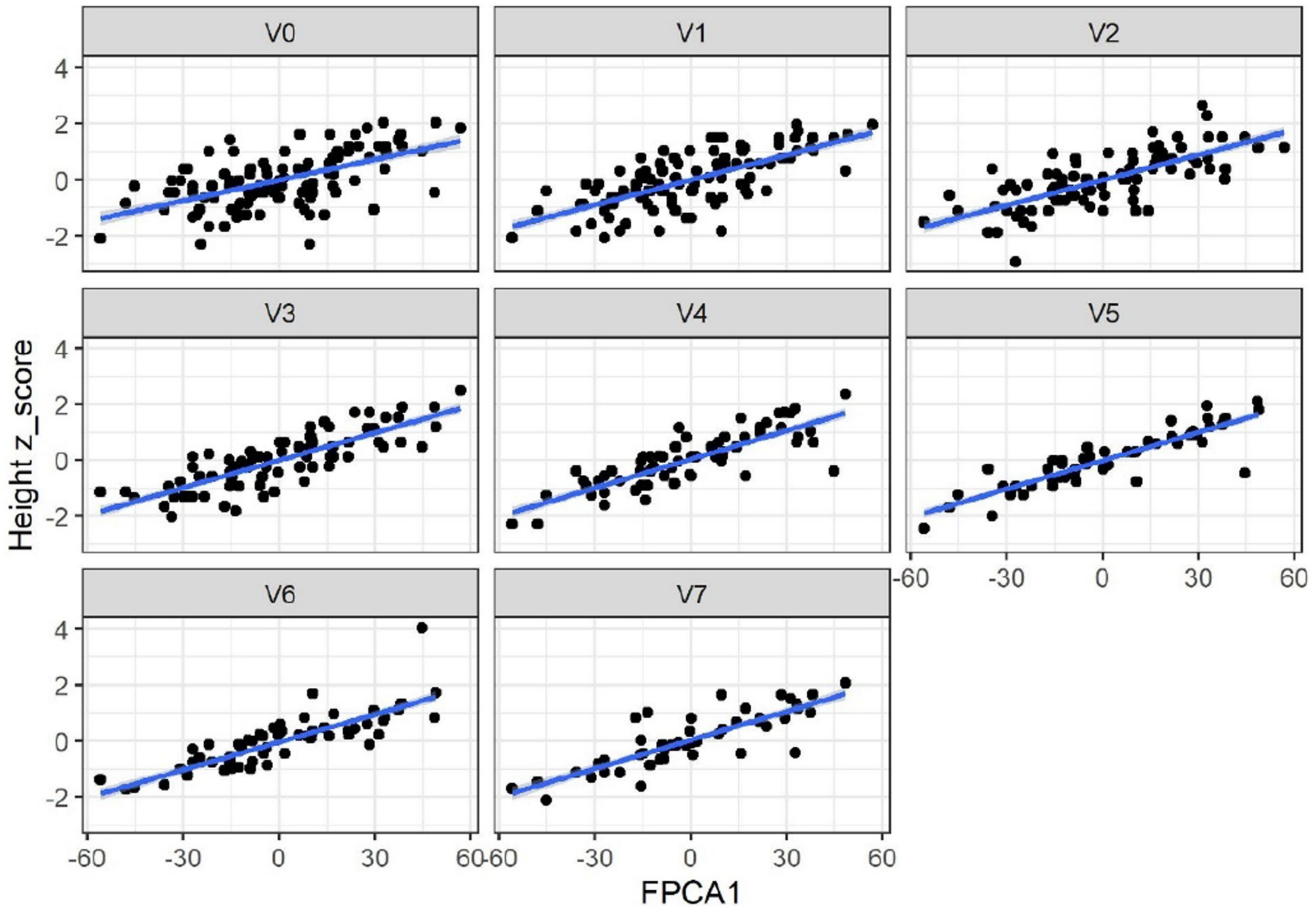
FPCA1 score is positively correlated with the z-scores for length (cm). Correlations: 0.54 (V0 = 2–5 wk), 0.70 (V1 = 6 wk), 0.73 (V2 = 3 mo), 0.82 (V3 = 6 mo), 0.81 (V4 = 9 mo), 0.88 (V5 = 12 mo), 0.88 (V6 = 18 mo), 0.78 (V7 = 24 mo). All *p*-values <0.01.

**Figure 7:**
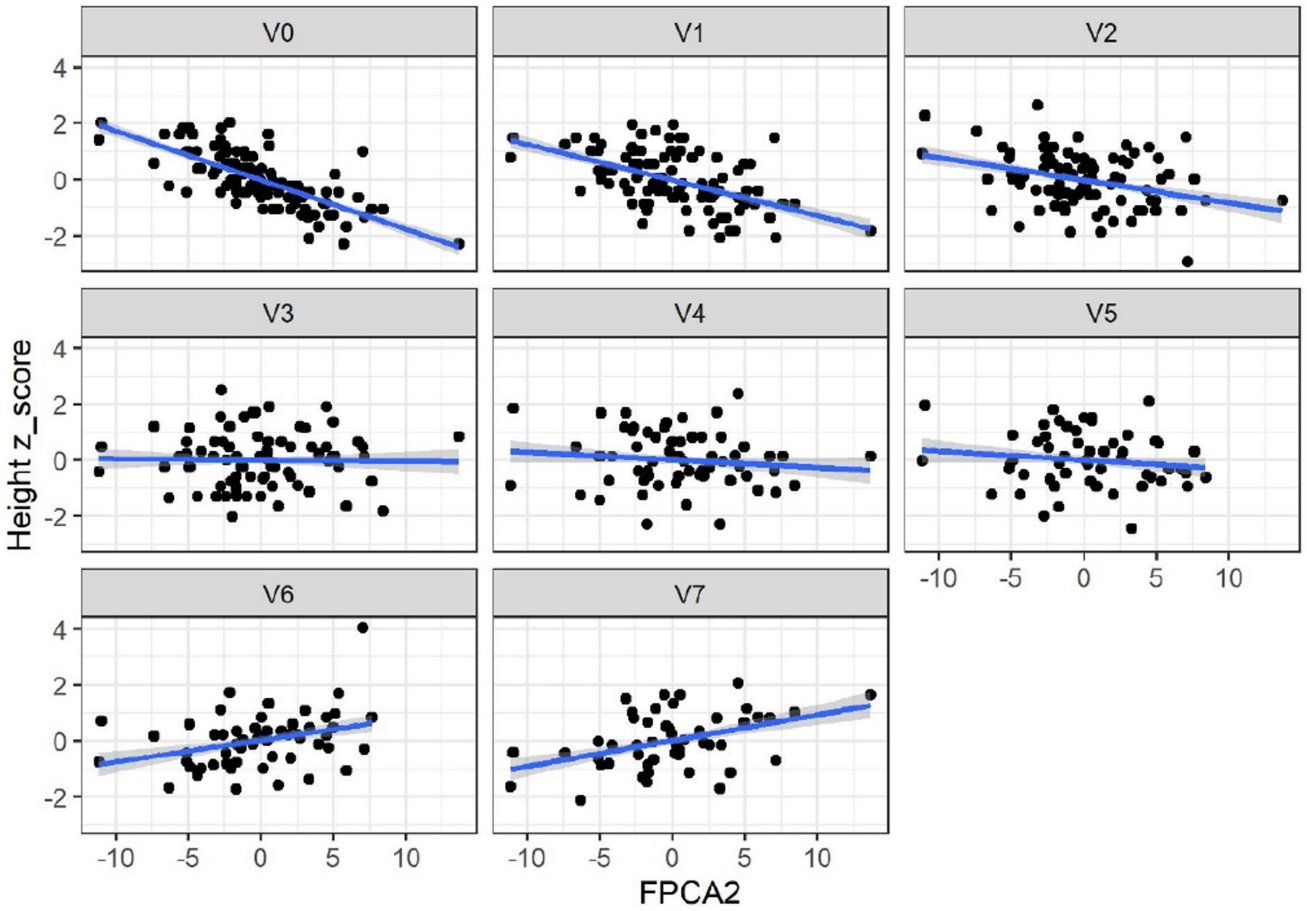
Correlations between FPCA2 scores and length (cm). Correlations: –0.79 (V0), –0.56 (V1), −0,37(V2), −0.05 (V3), −0.13 (V4), −0.12 (V5), 0.3 (V6), 0.35 (V7). Correlations were significant only at V0-V2 and V6-V7.

**Figure 8:**
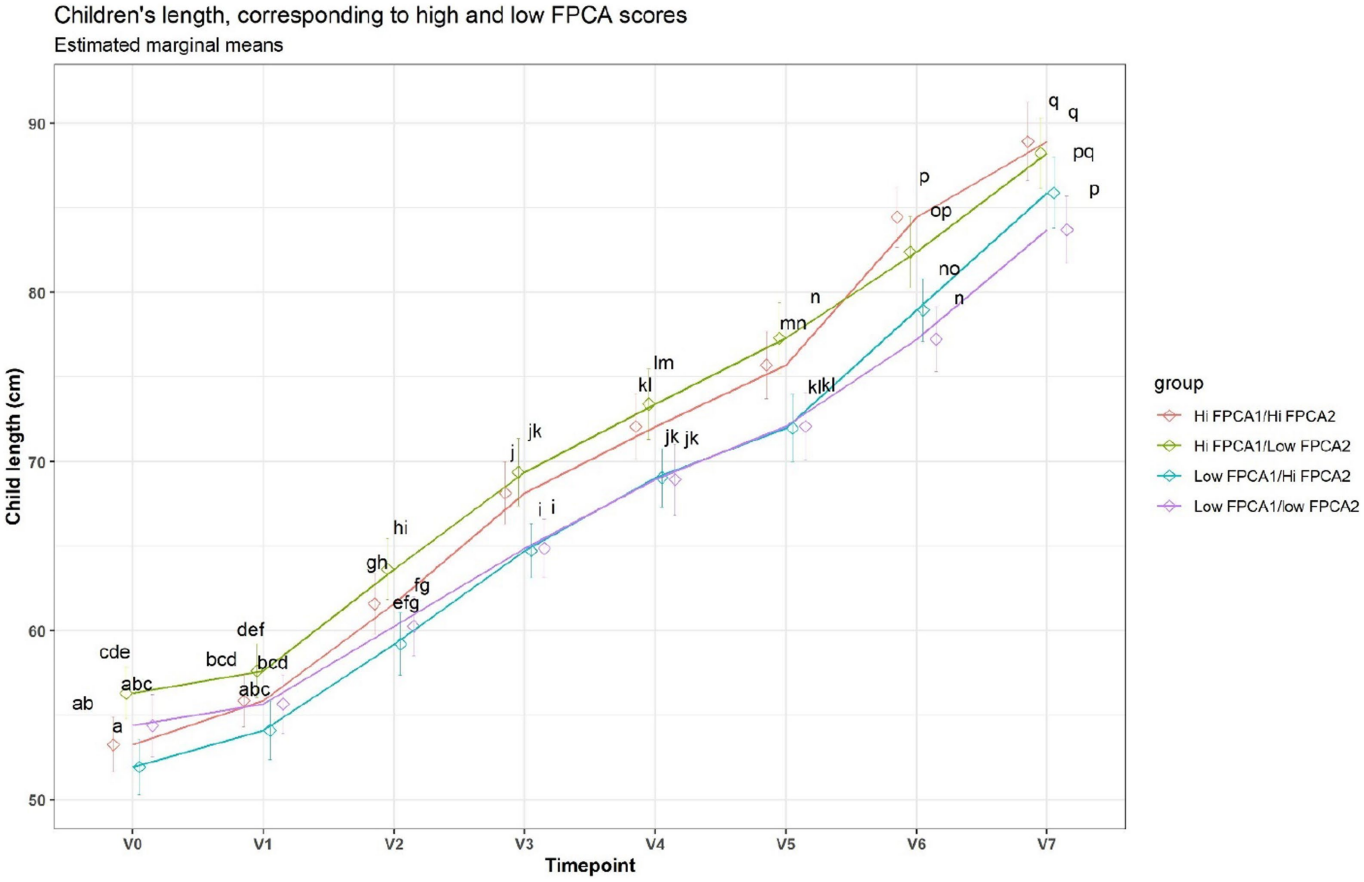
Average trajectories for length. Trajectories were split in 4 groups, based on median FPCA scores. A shared letter indicates a non-significant difference at the corresponding time point. For example, the ‘Low FPCA1l/high FPCA2’ is significantly lower than the ‘Hi FPCA1/low FPCA2’ at V0, V1, V2, V3, but not at V7.

When applied to the trajectories of body length, FPCA resulted in 97% of the variance explained by the first two scores, the first score FPCA1 explained 93% of the variance, and was positively correlated with the length z-scores at all time points (Figure 6). The second score (FPCA2) explained 4% of the variance; it was negatively correlated with length at early timepoints, and positively correlated at V7, so positive values of FPCA2 correspond to trajectories having a slow growth before V3 months and an increase at V6 and V7 (Figure 7). We will refer to FPCA1 as ‘general growth’ and to FPCA2 as ‘higher velocity’. See Figure 8 for an illustration of these patterns.

General growth was higher for boys (*T* test, *p* < 0.01), indicating that in girls most trajectories of length were below the trajectories for boys. Higher velocity rates were not significantly different between males and females.

**Figure 9:**
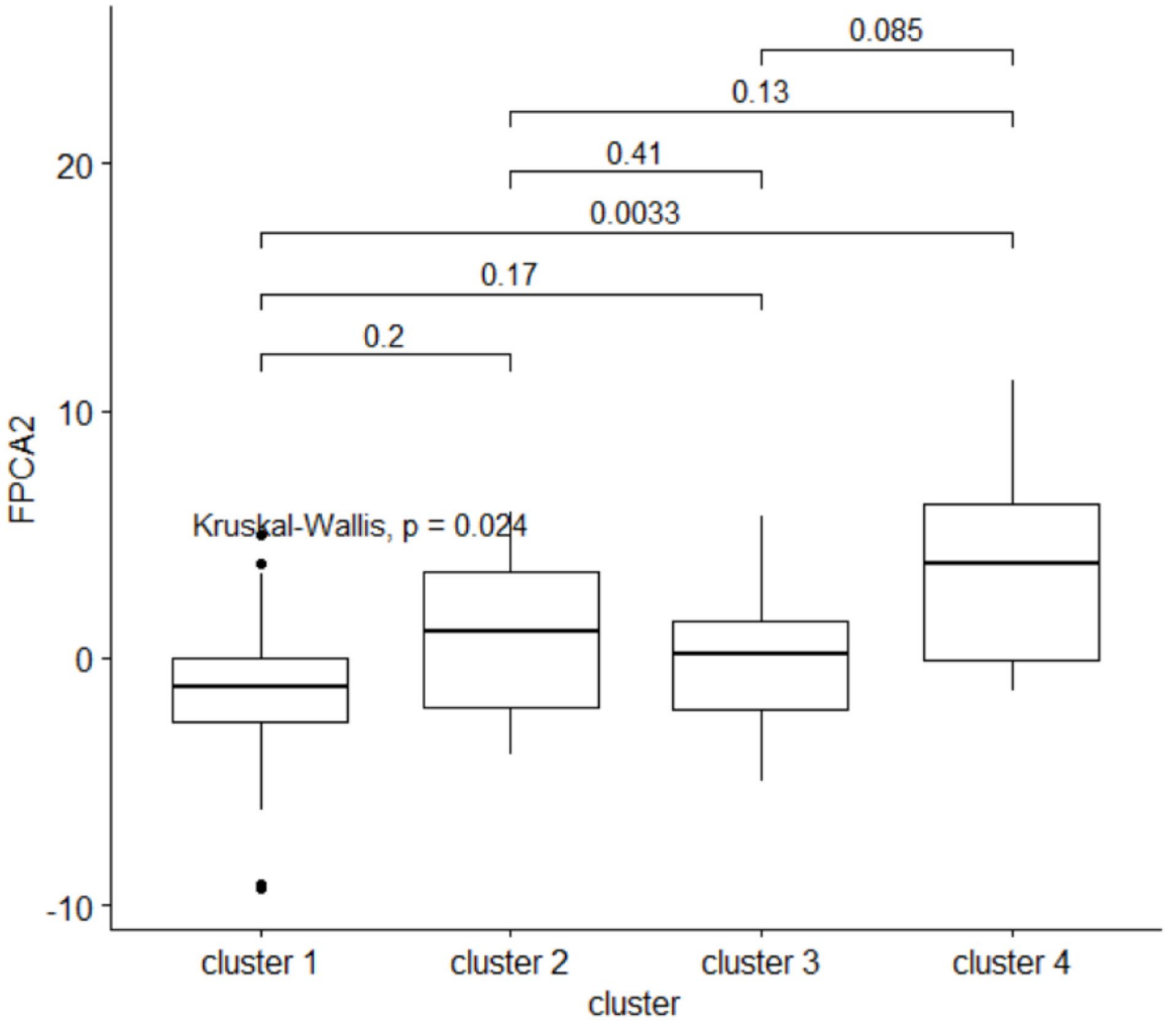
The second FPCA score for the child’s length is associated with the HMO clustering. Above the boxplots, are shown the *p*-values from a *post-hoc* Dunn pairwise comparison test, with multiplicity correction.

Clusters of HMOs at V2 were significantly associated with higher velocity (FPCA2 score), but not with general growth (FPCA1 score), with cluster 4 having significantly higher values than cluster 1 (Figure 9). The same trend was observed at V0 and V1, albeit not significant.

Concentrations of 3’GL, 3FL, 6’GL, DSNLT, LNFP-II, LNFP-III, LNT, LSTb were negatively associated with higher velocity (Supplementary Table 3). For 3FL, this negative association was consistent across the time of lactation (V0, V1, V2).

#### Weight

For the weight, the first score FPCA1 explained 91% of the variance, and was highly correlated with the weight z-scores (*ρ* = 0.58 at V0, 0.72 at V1, 078 at V2, 0.84 at V3, 0.9 at V4, 0.9 at V5, 0.93 at V6, 0.9 at V7, all significant). The second score FPCA2 explained 7% of the variance, and Spearman correlation coefficients with z-scores were − 0.5 at V0, −0.57 at V1, −0.59 at V2, −0.53 at V3, −0.37 at V4, all significant (*p* < 0.01); correlations at V5-V6 were not significant. At V7, there was a significant positive correlation between FPCA2 and the z-score (*ρ* = 0.21); higher values of FPCA2 correspond to weight trajectories that are below the average before 9 months and above the average afterwards. FPCA1 was positively and significantly correlated with growth velocity at V2, 3 months (*ρ* = 0.48); at later time points FPCA1 and velocity were not significantly correlated. FPCA2 was significantly positively correlated with velocity at V4, 9 months (*ρ* = 0.49) and V5, 12 months (*ρ* = 0.57).

As in the case of length, clusters of HMOs at V2 were significantly associated to higher velocity for weight, as measured by FPCA2 (Kruskal-Wallis, *p* = 0.02, average values were − 0.7 for clusters 1,2, −0.2 for cluster 3, 2.7 for cluster 4). A post-hoc Dunn comparison test resulted in significantly higher values in cluster 4 compared to the other clusters.

General growth (FPCA1) was higher for boys (*T* test, *p* = 0.02) with a mean value of 2.02 kg, versus a mean value of −1.73 for girls. FPCA2 was higher for girls (*T* test, *p* = 0.04), with a mean value of 0.33 kg, versus a mean value of −0.57 for boys.

#### Head circumference

For the head circumference, the first score FPCA1 explained 93% of the variance, and the second score FPCA2 explained 4% of the variance. Again, we can interpret FPCA1 as a measure of general growth and FPCA2 as a measure of higher velocity. FPCA scores for head circumference trajectories were not significantly associated with the HMO clustering.

## Discussion

Our analysis aimed to explore the temporal and interindividual variability of the HMOs during the first 3 months of lactation, and to investigate the potential effect on growth. Since several groups of HMOs are highly correlated, multivariate methods may help to understand their interactions, and their cumulative effects. In this exploratory analysis, we proposed a modelling methodology to overcome some of the difficulties that occur when analyzing such data, in particular to address at the same time the non-linearity of growth patterns and their potential association with multi-dimensional, highly correlated HMO concentrations.

We applied a data-driven algorithm to assign mothers to clusters sharing similar HMO profiles. The significance of this clustering is that it is based on the concentrations of a panel of 24 HMOs, and takes into account their mutual correlations. The clustering algorithm produced 4 clusters, distinct from the 4 milk types that are usually defined based on fucosyltransferase (*FUT2* and *FUT3*) polymorphisms. However, the two partitions overlap: for example, our clusters 1 and 2 were composed of samples from mothers belonging to the (FUT2^+^, FUT3^+^) group and they differed in their respective concentrations of several fucosylated HMOs, including 3FL (higher in cluster 1), LNnT (higher in cluster 2), DFL (higher in cluster 1). Therefore, clusters 1 and 2 split the (FUT2^+^, FUT3^+^) group in two subgroups, with distinct levels of several of the most abundant HMOs; this might reflect differences in FUT enzymatic activity with FUT3 having potentially a stronger activity in cluster 1 compared to cluster 2. Cluster 3 corresponded to the (FUT2^−^, FUT3^+^) type. Cluster 4 was analog to the milk group 3 (FUT2^+^, FUT3^−^) with the highest concentrations of 2’FL, DFLNHa, LNFP-I, and lowest concentrations of 3FL, LNDF-I, LNFP-II, LNFP-V.

Previous studies (15) reported that 3FL, LNFP-II and LNnFP-V had their highest concentration in the (FUT2^−^, FUT3^+^) group, similarly to what we observed in our cluster 3. Likewise, 2’FL, LNFP-I and DFLNHa were highest in the (FUT2^+^, FUT3^−^) group, corresponding to our cluster 4. These dynamics can be explained by the substrate and enzyme availability as well as the competition between FUT2 and FUT3 enzymes. Cluster 1 had a lower FUT2 activity compared to FUT3. The opposite is true for Cluster 2. When both enzymes are necessary to synthesize an HMO like in the case of LNDFH-I, more is synthesized in the cluster with a presumably stronger FUT2.

In general, the intra-individual variability was significantly lower compared to inter-individual variability. While subjects were consistently clustered in clusters 3 and 4, independently of the time of lactation, some subjects switched from cluster 2 to 1. This might be explained by a difference in gene regulation and enzyme activity over time, in clusters 1 and 2 with the same Se and Le status, which may follow different patterns of change for each HMO. This means that the overall breastmilk composition, while remaining in the (FUT2^+^, FUT3^+^) group, can vary over the lactation period, with some HMOs decreasing more rapidly than others, changing the ratios between HMOs. We therefore suggest that it may be important to distinguish these subgroups in a statistical analysis, as these different rates of change might have a biological relevance.

When HMOs were expressed as % of total measured HMO, comparing Figure 2 with Supplementary Figure 9, patterns look very similar for several HMOs, including 3FL and LNFP-I, but differences appear for 3’SL and other HMOs. This highlights the fact that the concentration of a specific HMO can increase over time while decreasing as % of total measured HMO.

Only a few studies to date have investigated variations in the HMO composition in relation to maternal age (35), but the results are in general not conclusive. Our clusters of HMO composition were not associated with maternal characteristics such as age and maternal ethnicity. The same lack of association was observed for the levels of 2’FL and LNFP-II and, a fortiori, for the clustering based on the FUT2, FUT3 levels.

A negative association of 3’SL with ppBMI had been reported by Saben et al. (36), who suggested that HMO sialylation may be negatively associated with maternal adiposity. According to Samuel et al. overweight women from a European cohort had significantly higher concentrations of 3′SL, 6′GL and DSLNT (at day 2), 6′SL (at day 17) and LNFP-V (at 3 and 4 months), while lower concentrations of LNnT (at day 2), LNT (at 1 and 3 months) and LNFP-V (at 2 months) compared to normal weight women (*p* < 0.05 for all). Other studies reported non-significant associations between HMO concentrations and ppBMI (37). We observed in our data that 6’GL, LNH and LSTc were consistently higher in the overweight group. To the best of our knowledge, associations of LNH and LSTc concentrations with ppBMI have not been reported before.

Samuel et al. reported lower concentrations of 2’FL, 3’SL and 6’GL at 2 days, among women delivering through C-section (15). Although we observed the same trend for these HMOs all time points, it was not significant, possibly because this association might be stronger at a very early stage of lactation.

It was proposed that HMO composition might affect child growth by altering the composition of the gut microbiome (38). Our results suggest a possible association of the Lewis negative status with a specific longitudinal growth pattern, as discussed below.

Higher HMO diversity and evenness at 1 month have been associated to lower total and percentage fat mass at 1mo (39). In Lagström et al. (40), the concentration of LNnT (at 3 months) was inversely associated and that of 2’FL (3 months) was directly associated with child length and weight z-scores in a model adjusted for maternal pre-pregnancy BMI, mode of delivery, birthweight z-score, sex and time. In an exploratory study, Sprenger et al. (16) found that FUT2 related alterations of breast milk HMOs composition as assessed through 2’FL concentrations, did not impact growth of breastfed infants during the first 4 months of life. However, they reported a non-statistically significant trend, with males from mothers with low 2’FL milk appearing to have a slightly higher BMI at 1 month, which was not seen any more at 4 months of age when they rather had a smaller BMI and body weight gain. Our approach suggests a complex, dynamic association between growth and HMO composition, illustrated by diverging trajectories starting from 3 months. However, these associations did not address the question of how changes in growth trajectories could be associated with early exposure to higher concentrations of HMO. Therefore, we analyzed the trajectories of length, weight, and head circumference over a period ranging from 2–5 weeks to 24 months, applying a data-driven approach derived from functional data analysis (23, 28, 29). In our study, each trajectory could be accurately described by two numbers (FPCA1, FPCA2), the first describing general growth (higher scores correspond to trajectories above the average at all time points), and the second describing a pattern of increased velocity at 12–18 months. These two scores jointly describe accurately the shape of the individual trajectories and can be therefore taken as a space of coordinates describing the growth trajectories.

Children fed with breast milk of cluster 4 (high in 2’FL, DFNHA, LNFP-I, LNDFH-I, low in 3FL) at 3 months, experienced a slight growth spurt after 12 months, compared to children fed with breast milk of cluster 1 (high in DFSL, LNDF-I). Since cluster 1 belongs to the (FUT2^+^, FUT3^+^) group and cluster 4 belongs to the (FUT2^+^, FUT3^−^) group, this can be interpreted as a possible association of the Lewis negative status with this particular longitudinal growth pattern. This supports the possibility that HMO concentrations should be looked at in combination when assessing associations with development, and that the non-linear patterns in the developmental trajectories must be appropriately modelled.

To the best of our knowledge, this is the first study to investigate the longitudinal association between clusters of HMO composition and child’s physical development. Among the limitations, we acknowledge the modest sample size and the limited age range 0–24 months, limiting the interpretation of the growth patterns to early growth.

Using longitudinal data, we introduced novel methodological approaches, like network analysis of compositional data and functional PCA, to address the multivariate nature of the data. This approach may help to reveal complex longitudinal patterns, and can help future studies in the field.

## Data availability statement

The raw data supporting the conclusions of this article will be made available by the authors, without undue reservation.

## Ethics statement

The studies involving humans were approved by Ethics boards at Rhode Island Hospital in Providence, RI, and Pennington Biomedical Research Center, in Baton Rouge, LA. The studies were conducted in accordance with the local legislation and institutional requirements. Written informed consent for participation in this study was provided by the participants’ legal guardians/next of kin.

## Author contributions

FM conducted the data analyses and wrote the manuscript draft. AB, PR, SA, SD, and NS reviewed the manuscript. SA performed the quantification of the HMOs. NS and SD designed the study. All authors contributed to the article and approved the submitted version.

## Footnotes

^1^ https://www.who.int/tools/child-growth-standards/standards
