# Supplementary material for: Human milk oligosaccharide composition and associations with growth: results from an observational study in the US
Source: Front Nutr. 2023 Oct 3;10:1239349. doi: 10.3389/fnut.2023.1239349 (PMC10580431; doi:10.3389/fnut.2023.1239349)
Supplement: Supplementary file 1 [file Table_1.docx]

| **Measure** | **Unit** | **Visit** | **mean** | **sd** | **min** | **max** | **N** |
| --- | --- | --- | --- | --- | --- | --- | --- |
| Length | cm | 2-4 weeks | 54.0 | 2.4 | 48.5 | 59.0 | 106 |
| Length | cm | 6 weeks | 55.9 | 2.1 | 51.5 | 60.0 | 100 |
| Length | cm | 3 months | 61.0 | 2.7 | 53.2 | 68.0 | 86 |
| Length | cm | 6 months | 66.7 | 2.8 | 61.0 | 73.7 | 76 |
| Length | cm | 9 months | 70.7 | 2.9 | 64.0 | 77.5 | 69 |
| Length | cm | 12 months | 74.0 | 3.3 | 66.0 | 81.0 | 60 |
| Length | cm | 18 months | 80.7 | 4.1 | 73.5 | 97.2 | 59 |
| Length | cm | 24 months | 86.5 | 3.0 | 80.0 | 92.7 | 54 |
| Weight | Kg | 2-4 weeks | 4.2 | 0.5 | 2.9 | 5.6 | 106 |
| Weight | Kg | 6 weeks | 4.7 | 0.6 | 3.4 | 6.1 | 100 |
| Weight | Kg | 3 months | 5.9 | 0.8 | 4.5 | 7.6 | 86 |
| Weight | Kg | 6 months | 7.5 | 1.0 | 5.8 | 9.8 | 76 |
| Weight | Kg | 9 months | 8.7 | 1.0 | 6.4 | 11.0 | 69 |
| Weight | kg | 12 months | 9.4 | 1.0 | 7.3 | 11.8 | 60 |
| Weight | Kg | 18 months | 11.1 | 1.1 | 9.1 | 13.6 | 59 |
| Weight | kg | 24 months | 12.4 | 1.4 | 9.7 | 15.4 | 54 |

Supplementary table 1. Length and weight, at each time point. Only breastfed children included. N=number of children measured.
