# Supplementary material for: Human milk oligosaccharide composition and associations with growth: results from an observational study in the US
Source: Front Nutr. 2023 Oct 3;10:1239349. doi: 10.3389/fnut.2023.1239349 (PMC10580431; doi:10.3389/fnut.2023.1239349)
Supplement: Supplementary file 2 [file Table_2.docx]

| **HMO** | **VISIT** | **MIN** | **MEDIAN** | **MEAN** | **MAX** | **N** |
| --- | --- | --- | --- | --- | --- | --- |
| 2'-FUCOSYLLACTOSE | 2-5 wk | 0.0 | 2,038.5 | 2,051.8 | 7,394.9 | 106 |
|  | 6 wk | 0.0 | 1,552.6 | 1,826.6 | 4,055.3 | 76 |
|  | 3 mo | 0.0 | 1,905.2 | 1,437.2 | 7,358.2 | 97 |
| 3'-GALACTOSYLLACTOSE | 2-5 wk | 1.8 | 5.3 | 8.2 | 156.6 | 106 |
|  | 6 wk | 1.8 | 4.6 | 6.0 | 39.9 | 97 |
|  | 3 mo | 1.4 | 2.8 | 3.6 | 18.4 | 76 |
| 3'-SIALYLLACTOSE | 2-5 wk | 78.5 | 150.1 | 156.1 | 290.3 | 106 |
|  | 6 wk | 71.8 | 148.1 | 151.6 | 295.0 | 97 |
|  | 3 mo | 72.2 | 131.5 | 141.8 | 283.8 | 76 |
| 3-FUCOSYLLACTOSE | 2-5 wk | 4.2 | 591.0 | 710.5 | 2,891.5 | 106 |
|  | 6 wk | 4.8 | 780.9 | 871.8 | 3,069.9 | 97 |
|  | 3 mo | 122.7 | 1,043.1 | 1,118.1 | 3,584.9 | 76 |
| 3,2'-DIFUCOSYLLACTOSE | 2-5 wk | 0.0 | 236.1 | 304.0 | 3,750.8 | 106 |
|  | 6 wk | 0.0 | 247.8 | 302.9 | 1,478.7 | 97 |
|  | 3 mo | 0.0 | 236.7 | 269.1 | 1,677.9 | 76 |
| 6'-GALACTOSYLLACTOSE | 2-5 wk | 0.0 | 29.7 | 32.0 | 95.8 | 106 |
|  | 6 wk | 8.2 | 22.4 | 24.7 | 86.0 | 97 |
|  | 3 mo | 6.8 | 13.2 | 15.1 | 42.0 | 76 |
| 6'-SIALYLLACTOSE | 2-5 wk | 107.8 | 460.8 | 504.5 | 1,114.4 | 106 |
|  | 6 wk | 75.0 | 323.2 | 330.7 | 962.6 | 97 |
|  | 3 mo | 18.1 | 124.6 | 139.1 | 367.5 | 76 |
| A-TETRASACCHARIDE | 2-5 wk | 7.5 | 7.5 | 42.0 | 304.0 | 107 |
|  | 6 wk | 7.5 | 7.5 | 38.2 | 314.0 | 97 |
|  | 3 mo | 7.5 | 7.5 | 38.4 | 354.0 | 76 |
| DIFUCOSYLLACTO-N-HEXAOSE-A | 2-5 wk | 0.0 | 113.4 | 163.1 | 896.4 | 106 |
|  | 6 wk | 0.0 | 67.0 | 107.6 | 777.6 | 97 |
|  | 3 mo | 0.0 | 27.5 | 56.1 | 425.1 | 76 |
| DISIALYLLACTO-N-TETRAOSE | 2-5 wk | 0.0 | 314.7 | 309.4 | 773.0 | 106 |
|  | 6 wk | 2.4 | 207.3 | 235.0 | 1,000.4 | 97 |
|  | 3 mo | 3.8 | 123.0 | 127.3 | 426.5 | 76 |
| Hex4 HexNAc2 (2) | 2-5 wk | 0.0 | 20.2 | 27.2 | 246.5 | 106 |
|  | 6 wk | 0.0 | 18.6 | 25.2 | 244.7 | 97 |
|  | 3 mo | 0.0 | 8.9 | 13.7 | 66.5 | 76 |
| LACTO-N-DIFUCOHEXAOSE-I | 2-5 wk | 0.0 | 904.8 | 835.0 | 2,587.2 | 106 |
|  | 6 wk | 0.0 | 846.1 | 759.8 | 2,819.6 | 97 |
|  | 3 mo | 0.0 | 624.5 | 541.1 | 1,755.2 | 76 |
| LACTO-N-FUCOPENTAOSE-I | 2-5 wk | 0.0 | 741.0 | 931.7 | 4,164.2 | 106 |
|  | 6 wk | 0.0 | 456.9 | 619.4 | 3,029.5 | 97 |
|  | 3 mo | 0.0 | 266.5 | 383.8 | 2,476.8 | 76 |
| LACTO-N-FUCOPENTAOSE-II | 2-5 wk | 0.0 | 424.1 | 535.5 | 2,253.8 | 105 |
|  | 6 wk | 0.0 | 408.2 | 512.5 | 2,020.3 | 96 |
|  | 3 mo | 0.0 | 356.9 | 433.8 | 2,040.7 | 76 |
| LACTO-N-FUCOPENTAOSE-III | 2-5 wk | 0.0 | 347.8 | 342.4 | 624.9 | 106 |
|  | 6 wk | 25.3 | 386.6 | 378.8 | 673.3 | 97 |
|  | 3 mo | 140.0 | 368.6 | 378.4 | 652.3 | 76 |
| LACTO-N-FUCOPENTAOSE-V | 2-5 wk | 1.4 | 56.5 | 86.4 | 375.0 | 106 |
|  | 6 wk | 0.0 | 51.5 | 76.6 | 348.1 | 97 |
|  | 3 mo | 3.7 | 43.1 | 59.5 | 263.3 | 76 |
| LACTO-N-HEXAOSE | 2-5 wk | 20.0 | 58.5 | 72.3 | 260.7 | 106 |
|  | 6 wk | 13.1 | 41.4 | 49.7 | 143.7 | 97 |
|  | 3 mo | 0.0 | 23.4 | 28.5 | 116.1 | 76 |
| LACTO-N-NEODIFUCOHEXAOSE | 2-5 wk | 0.0 | 15.6 | 23.2 | 288.4 | 105 |
|  | 6 wk | 0.0 | 16.2 | 22.3 | 188.2 | 97 |
|  | 3 mo | 0.0 | 11.6 | 16.3 | 127.6 | 76 |
| LACTO-N-NEOFUCOPENTAOSE-V | 2-5 wk | 0.0 | 12.0 | 12.7 | 47.2 | 106 |
|  | 6 wk | 0.0 | 13.3 | 14.8 | 63.8 | 97 |
|  | 3 mo | 0.0 | 10.9 | 12.7 | 59.3 | 76 |
| LACTO-N-NEOTETRAOSE | 2-5 wk | 0.0 | 147.2 | 158.9 | 509.0 | 106 |
|  | 6 wk | 0.0 | 141.8 | 144.9 | 452.6 | 97 |
|  | 3 mo | 12.0 | 97.9 | 107.8 | 299.4 | 76 |
| LACTO-N-TETRAOSE | 2-5 wk | 61.5 | 1,018.9 | 1,197.3 | 4,000.9 | 106 |
|  | 6 wk | 94.3 | 821.0 | 897.9 | 2,670.2 | 97 |
|  | 3 mo | 46.6 | 548.4 | 657.6 | 1,845.6 | 76 |
| MONOFUCOSYLLACTO-N-HEXAOSE-III | 2-5 wk | 25.9 | 346.3 | 383.3 | 1,497.8 | 106 |
|  | 6 wk | 26.0 | 225.4 | 263.1 | 871.7 | 97 |
|  | 3 mo | 7.4 | 120.6 | 138.7 | 467.2 | 76 |
| SIALYLLACTO-N-TETRAOSE-B | 2-5 wk | 7.1 | 78.6 | 92.2 | 1,422.6 | 106 |
|  | 6 wk | 21.4 | 72.0 | 100.8 | 2,100.0 | 97 |
|  | 3 mo | 10.7 | 56.2 | 67.1 | 750.4 | 76 |
| SIALYLLACTO-N-TETRAOSE-C | 2-5 wk | 38.6 | 166.1 | 191.1 | 572.4 | 106 |
|  | 6 wk | 17.4 | 107.8 | 116.0 | 314.0 | 97 |
|  | 3 mo | 0.0 | 35.1 | 43.2 | 175.9 | 76 |
|  |  |  |  |  |  |  |

Supplementary table 2. Descriptive statistics for the concentrations of HMOs at each time point.
