# Supplementary material for: Human milk oligosaccharide composition and associations with growth: results from an observational study in the US
Source: Front Nutr. 2023 Oct 3;10:1239349. doi: 10.3389/fnut.2023.1239349 (PMC10580431; doi:10.3389/fnut.2023.1239349)
Supplement: Supplementary file 3 [file Table_3.docx]

| **HMO** | **VISIT** | **cluster 1** | **cluster 2** | **cluster 3** | **cluster 4** | |
| --- | --- | --- | --- | --- | --- | --- |
| 2'FL | V0 | 1730.3 | 2642.3 | 35.9 | 3958.7 |  |
| 2'FL | V1 | 1667.8 | 2639.9 | 6.8 | 3656.7 |  |
| 2'FL | V2 | 1563.6 | 2563.5 | 6.5 | 2771.5 |  |
| 3'GL | V0 | 6.3 | 5.4 | 6.4 | 19.4 |  |
| 3'GL | V1 | 6.8 | 5.4 | 4.7 | 7.3 |  |
| 3'GL | V2 | 4.8 | 4.5 | 4.0 | 4.7 |  |
| 3'SL | V0 | 160.0 | 155.7 | 137.1 | 178.8 |  |
| 3'SL | V1 | 159.6 | 146.6 | 128.5 | 184.9 |  |
| 3'SL | V2 | 144.7 | 143.7 | 126.6 | 161.3 |  |
| 3FL | V0 | 881.4 | 469.5 | 1302.0 | 162.5 |  |
| 3FL | V1 | 1031.1 | 495.2 | 1477.9 | 191.6 |  |
| 3FL | V2 | 1150.6 | 484.8 | 1739.1 | 235.3 |  |
| 6'GL | V0 | 28.3 | 30.4 | 34.3 | 36.7 |  |
| 6'GL | V1 | 24.8 | 23.5 | 24.6 | 27.1 |  |
| 6'GL | V2 | 15.4 | 15.8 | 13.4 | 16.0 |  |
| 6'SL | V0 | 467.6 | 489.3 | 535.3 | 587.7 |  |
| 6'SL | V1 | 315.5 | 327.7 | 330.7 | 389.4 |  |
| 6'SL | V2 | 119.1 | 146.9 | 155.2 | 202.8 |  |
| DFNHA | V0 | 66.9 | 209.3 | 19.4 | 491.3 |  |
| DFNHA | V1 | 52.8 | 157.6 | 17.6 | 350.0 |  |
| DFNHA | V2 | 28.0 | 99.7 | 16.5 | 290.8 |  |
| DFSL | V0 | 491.7 | 281.0 | 22.5 | 490.1 |  |
| DFSL | V1 | 492.1 | 275.9 | 22.5 | 336.1 |  |
| DFSL | V2 | 413.1 | 248.8 | 22.5 | 143.3 |  |
| DSNLT | V0 | 320.2 | 274.2 | 391.0 | 249.2 |  |
| DSNLT | V1 | 269.0 | 180.7 | 255.1 | 240.9 |  |
| DSNLT | V2 | 128.5 | 105.1 | 136.6 | 127.7 |  |
| HEX | V0 | 15.2 | 41.0 | 12.4 | 37.8 |  |
| HEX | V1 | 19.2 | 38.5 | 12.0 | 40.7 |  |
| HEX | V2 | 13.2 | 32.7 | 11.5 | 11.4 |  |
| LNDF-I | V0 | 1411.0 | 1185.5 | 27.3 | 11.2 |  |
| LNDF-I | V1 | 1265.7 | 962.6 | 5.5 | 19.1 |  |
| LNDF-I | V2 | 831.0 | 701.5 | 5.5 | 5.0 |  |
| LNFP-I | V0 | 590.3 | 1169.1 | 61.1 | 2355.8 |  |
| LNFP-I | V1 | 406.3 | 911.0 | 13.5 | 1763.0 |  |
| LNFP-I | V2 | 318.5 | 792.7 | 13.5 | 1168.3 |  |
| LNFP-II | V0 | 632.9 | 340.5 | 1064.9 | 29.8 |  |
| LNFP-II | V1 | 610.2 | 252.7 | 934.9 | 51.4 |  |
| LNFP-II | V2 | 405.2 | 168.6 | 818.5 | 17.5 |  |
| LNFP-III | V0 | 385.0 | 298.2 | 395.5 | 284.2 |  |
| LNFP-III | V1 | 429.2 | 316.7 | 415.7 | 295.5 |  |
| LNFP-III | V2 | 383.5 | 301.7 | 419.0 | 353.2 |  |
| LNFP-V | V0 | 72.9 | 44.2 | 208.1 | 27.0 |  |
| LNFP-V | V1 | 72.0 | 30.6 | 172.4 | 28.3 |  |
| LNFP-V | V2 | 43.8 | 21.2 | 130.3 | 17.9 |  |
| LNH | V0 | 51.7 | 90.6 | 71.4 | 68.9 |  |
| LNH | V1 | 41.6 | 63.1 | 49.1 | 44.1 |  |
| LNH | V2 | 23.7 | 41.4 | 33.4 | 25.7 |  |
| LNnDFH | V0 | 19.2 | 22.9 | 19.6 | 31.7 |  |
| LNnDFH | V1 | 23.1 | 22.8 | 22.4 | 29.4 |  |
| LNnDFH | V2 | 23.2 | 19.7 | 14.0 | 16.0 |  |
| LNnFP-V | V0 | 17.4 | 10.7 | 13.5 | 10.3 |  |
| LNnFP-V | V1 | 20.6 | 10.3 | 13.4 | 9.5 |  |
| LNnFP-V | V2 | 14.6 | 9.5 | 12.2 | 9.5 |  |
| LNnT | V0 | 154.7 | 199.4 | 88.2 | 165.5 |  |
| LNnT | V1 | 155.9 | 192.6 | 70.6 | 126.9 |  |
| LNnT | V2 | 114.3 | 186.9 | 63.6 | 65.0 |  |
| LNT | V0 | 1062.9 | 1042.2 | 1828.6 | 775.6 |  |
| LNT | V1 | 880.1 | 742.8 | 1310.8 | 600.6 |  |
| LNT | V2 | 565.9 | 578.2 | 953.2 | 548.5 |  |
| LSTb | V0 | 93.4 | 69.6 | 90.9 | 180.3 |  |
| LSTb | V1 | 93.5 | 62.4 | 88.8 | 270.0 |  |
| LSTb | V2 | 71.9 | 54.6 | 69.4 | 49.9 |  |
| LSTc | V0 | 197.1 | 193.4 | 165.8 | 208.5 |  |
| LSTc | V1 | 118.8 | 124.7 | 92.2 | 122.7 |  |
| LSTc | V2 | 41.1 | 55.6 | 39.2 | 47.5 |  |
| MFLN-III | V0 | 218.2 | 358.5 | 616.9 | 373.2 |  |
| MFLN-III | V1 | 185.5 | 258.5 | 398.1 | 284.4 |  |
| MFLN-III | V2 | 92.5 | 149.1 | 214.7 | 198.8 |  |
|  |  |  |  |  |  | |

| **Coefficient** | **Estimate** | **std.error** | **statistic** | **p.value** | **VISIT** | **HMO** |
| --- | --- | --- | --- | --- | --- | --- |
| (Intercept) | 3.679 | 1.500 | 2.453 | 0.017 | V2 | 3'GL |
| HMO concentration | -0.668 | 0.320 | -2.090 | 0.040 |  |  |
| SEX [Male] | 0.234 | 0.371 | 0.630 | 0.531 |  |  |
| cluster1 | -3.725 | 1.636 | -2.277 | 0.026 |  |  |
| cluster2 | 0.528 | 2.354 | 0.224 | 0.823 |  |  |
| cluster3 | -1.318 | 0.708 | -1.862 | 0.067 |  |  |
| HMO concentration:cluster1 | 0.395 | 0.338 | 1.166 | 0.248 |  |  |
| HMO concentration:cluster2 | -0.096 | 0.481 | -0.199 | 0.843 |  |  |
| (Intercept) | 1.872 | 0.728 | 2.572 | 0.012 | V0 | 3FL |
| HMO concentration | -0.005 | 0.002 | -2.838 | 0.006 |  |  |
| SEX [Male] | 0.164 | 0.311 | 0.529 | 0.598 |  |  |
| cluster1 | 0.423 | 1.471 | 0.288 | 0.774 |  |  |
| cluster2 | 0.758 | 1.206 | 0.629 | 0.531 |  |  |
| cluster3 | -3.448 | 1.176 | -2.932 | 0.004 |  |  |
| HMO concentration:cluster1 | 0.002 | 0.002 | 1.104 | 0.272 |  |  |
| HMO concentration:cluster2 | -0.002 | 0.003 | -0.770 | 0.443 |  |  |
| HMO concentration:cluster3 | 0.006 | 0.002 | 3.215 | 0.002 |  |  |
| (Intercept) | 2.068 | 0.818 | 2.528 | 0.013 | V1 | 3FL |
| HMO concentrtion | -0.005 | 0.002 | -3.118 | 0.002 |  |  |
| SEX [Male] | 0.135 | 0.325 | 0.415 | 0.679 |  |  |
| cluster1 | -1.729 | 1.616 | -1.070 | 0.288 |  |  |
| cluster2 | 1.467 | 1.360 | 1.079 | 0.284 |  |  |
| cluster3 | -2.794 | 1.319 | -2.118 | 0.037 |  |  |
| HMO concentration:cluster1 | 0.005 | 0.002 | 2.249 | 0.027 |  |  |
| HMO concentration:cluster2 | -0.004 | 0.003 | -1.320 | 0.190 |  |  |
| HMO concentration:cluster3 | 0.006 | 0.002 | 3.091 | 0.003 |  |  |
| (Intercept) | 3.421 | 1.470 | 2.328 | 0.023 | V2 | 3FL |
| HMO concentration | -0.012 | 0.005 | -2.424 | 0.018 |  |  |
| SEX [Male] | 0.386 | 0.376 | 1.026 | 0.309 |  |  |
| cluster1 | -5.726 | 1.906 | -3.004 | 0.004 |  |  |
| cluster2 | 0.368 | 2.399 | 0.153 | 0.879 |  |  |
| cluster3 | -4.903 | 1.829 | -2.681 | 0.009 |  |  |
| HMO concentration:cluster1 | 0.013 | 0.005 | 2.545 | 0.013 |  |  |
| HMO concentration:cluster2 | 0.006 | 0.006 | 0.918 | 0.362 |  |  |
| HMO concentration:cluster3 | 0.013 | 0.005 | 2.552 | 0.013 |  |  |
| (Intercept) | 4.659 | 1.471 | 3.168 | 0.002 | V2 | 6'GL |
| HMO concentration | -0.255 | 0.090 | -2.821 | 0.006 |  |  |
| SEX [Male] | 0.163 | 0.369 | 0.442 | 0.660 |  |  |
| cluster1 | -4.919 | 1.668 | -2.949 | 0.004 |  |  |
| cluster2 | 1.352 | 2.913 | 0.464 | 0.644 |  |  |
| cluster3 | -3.450 | 2.073 | -1.664 | 0.101 |  |  |
| HMO concentration:cluster1 | 0.184 | 0.101 | 1.810 | 0.075 |  |  |
| HMO concentration:cluster2 | -0.079 | 0.177 | -0.448 | 0.656 |  |  |
| HMO concentration:cluster3 | 0.143 | 0.136 | 1.046 | 0.299 |  |  |
| (Intercept) | 2.734 | 1.111 | 2.460 | 0.016 | V2 | DSNLT |
| HMO concentration | -0.019 | 0.009 | -2.046 | 0.045 |  |  |
| SEX [Male] | 0.310 | 0.363 | 0.854 | 0.396 |  |  |
| cluster1 | -4.992 | 1.337 | -3.733 | 0.000 |  |  |
| cluster2 | 4.251 | 2.758 | 1.541 | 0.128 |  |  |
| cluster3 | -2.676 | 1.664 | -1.608 | 0.113 |  |  |
| HMO concentration:cluster1 | 0.026 | 0.011 | 2.438 | 0.017 |  |  |
| HMO concentration:cluster2 | -0.041 | 0.025 | -1.639 | 0.106 |  |  |
| HMO concentration:cluster3 | 0.016 | 0.012 | 1.292 | 0.201 |  |  |
| (Intercept) | 1.170 | 0.632 | 1.850 | 0.068 | V1 | LNFP-II |
| HMO concentration | -0.006 | 0.003 | -2.058 | 0.043 |  |  |
| SEX [Male] | 0.154 | 0.337 | 0.457 | 0.649 |  |  |
| cluster1 | -0.528 | 1.014 | -0.520 | 0.604 |  |  |
| cluster2 | -0.204 | 1.235 | -0.166 | 0.869 |  |  |
| cluster3 | -1.555 | 1.081 | -1.439 | 0.154 |  |  |
| HMO concentration:cluster1 | 0.005 | 0.003 | 1.453 | 0.150 |  |  |
| HMO concentration:cluster2 | -0.002 | 0.005 | -0.357 | 0.722 |  |  |
| HMO concentration:cluster3 | 0.006 | 0.003 | 2.010 | 0.048 |  |  |
| (Intercept) | 3.969 | 1.722 | 2.305 | 0.024 | V2 | LNFP-III |
| HMO concentration | -0.010 | 0.005 | -2.035 | 0.046 |  |  |
| SEX [Male] | 0.292 | 0.373 | 0.781 | 0.438 |  |  |
| cluster1 | -3.788 | 2.238 | -1.693 | 0.095 |  |  |
| cluster2 | -1.785 | 2.695 | -0.662 | 0.510 |  |  |
| cluster3 | -5.786 | 2.591 | -2.233 | 0.029 |  |  |
| HMO concentration:cluster1 | 0.006 | 0.006 | 0.931 | 0.355 |  |  |
| HMO concentration:cluster2 | 0.005 | 0.008 | 0.594 | 0.554 |  |  |
| HMO concentration:cluster3 | 0.013 | 0.006 | 2.033 | 0.046 |  |  |
| (Intercept) | 2.334 | 0.901 | 2.591 | 0.012 | V2 | LNT |
| HMO concentration | -0.003 | 0.001 | -2.197 | 0.031 |  |  |
| SEX [Male] | 0.231 | 0.368 | 0.629 | 0.532 |  |  |
| cluster1 | -5.676 | 1.219 | -4.656 | 0.000 |  |  |
| cluster2 | 1.378 | 1.680 | 0.820 | 0.415 |  |  |
| cluster3 | -2.131 | 1.429 | -1.492 | 0.140 |  |  |
| HMO concentration:cluster1 | 0.006 | 0.002 | 3.409 | 0.001 |  |  |
| HMO concentration:cluster2 | -0.002 | 0.003 | -0.893 | 0.375 |  |  |
| HMO concentration:cluster3 | 0.002 | 0.002 | 1.399 | 0.166 |  |  |
| (Intercept) | 2.384 | 0.884 | 2.696 | 0.009 | V2 | LSTb |
| HMO concentration | -0.032 | 0.013 | -2.403 | 0.019 |  |  |
| SEX [Male] | 0.181 | 0.374 | 0.484 | 0.630 |  |  |
| cluster1 | -3.821 | 0.974 | -3.923 | 0.000 |  |  |
| cluster2 | 2.204 | 1.826 | 1.207 | 0.232 |  |  |
| cluster3 | -2.078 | 1.617 | -1.285 | 0.203 |  |  |
| HMO concentration:cluster1 | 0.033 | 0.014 | 2.410 | 0.019 |  |  |
| HMO concentration:cluster2 | -0.039 | 0.030 | -1.316 | 0.193 |  |  |
| HMO concentration:cluster3 | 0.023 | 0.022 | 1.024 | 0.309 |  |  |

Supplementary table 3. Results from linear models with FPCA2 scores for Length as response variable and HMO concentration as independent variable, with an interaction term between HMO concentration and clustering, and adjusted for sex.
