# Supplementary material for: Human milk oligosaccharide composition and associations with growth: results from an observational study in the US
Source: Front Nutr. 2023 Oct 3;10:1239349. doi: 10.3389/fnut.2023.1239349 (PMC10580431; doi:10.3389/fnut.2023.1239349)
Supplement: Supplementary file 5 [file Image_2.pdf]

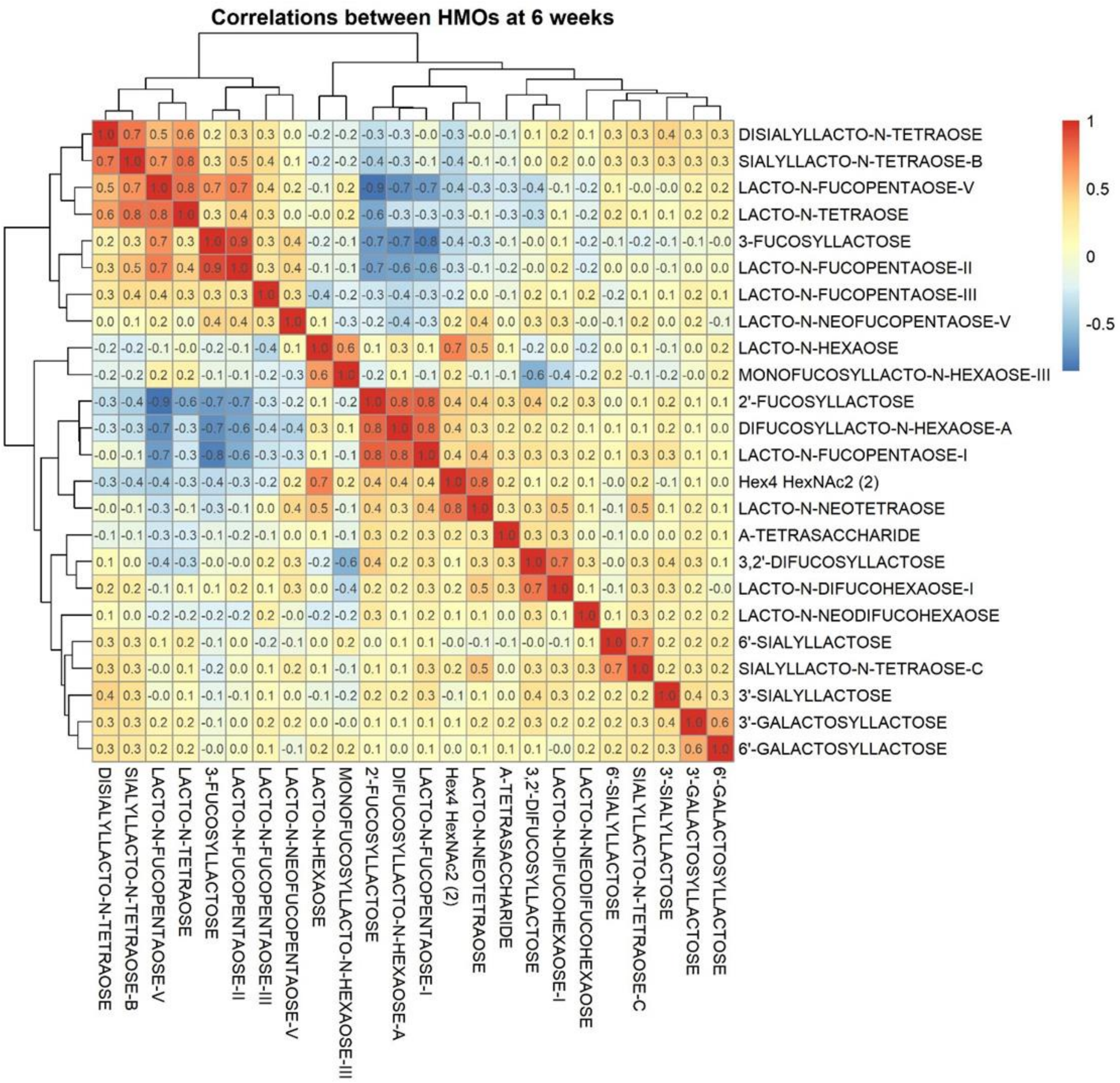
Correlations between HMOs at 6 weeks
DISIALYLLACTO-N-TETRAOSE
SIALYLLACTO-N-TETRAOSE-B
LACTO-N-FUCOPENTAOSE-V
LACTO-N-TETRAOSE
3-FUCOSYLLACTOSE
LACTO-N-FUCOPENTAOSE-II
LACTO-N-FUCOPENTAOSE-III
LACTO-N-NEOFUCOPENTAOSE-V
LACTO-N-HEXAOSE
MONOFUCOSYLLACTO-N-HEXAOSE-III
2'-FUCOSYLLACTOSE
DIFUCOSYLLACTO-N-HEXAOSE-A
LACTO-N-FUCOPENTAOSE-I
Hex4 HexNAc2 (2)
LACTO-N-NEOTETRAOSE
A-TETRASACCHARIDE
3,2'-DIFUCOSYLLACTOSE
LACTO-N-DIFUCOHEXAOSE-I
LACTO-N-NEODIFUCOHEXAOSE
6'-SIALYLLACTOSE
SIALYLLACTO-N-TETRAOSE-C
3'-SIALYLLACTOSE
3'-GALACTOSYLLACTOSE
6'-GALACTOSYLLACTOSE
1
0.5
0
-0.5

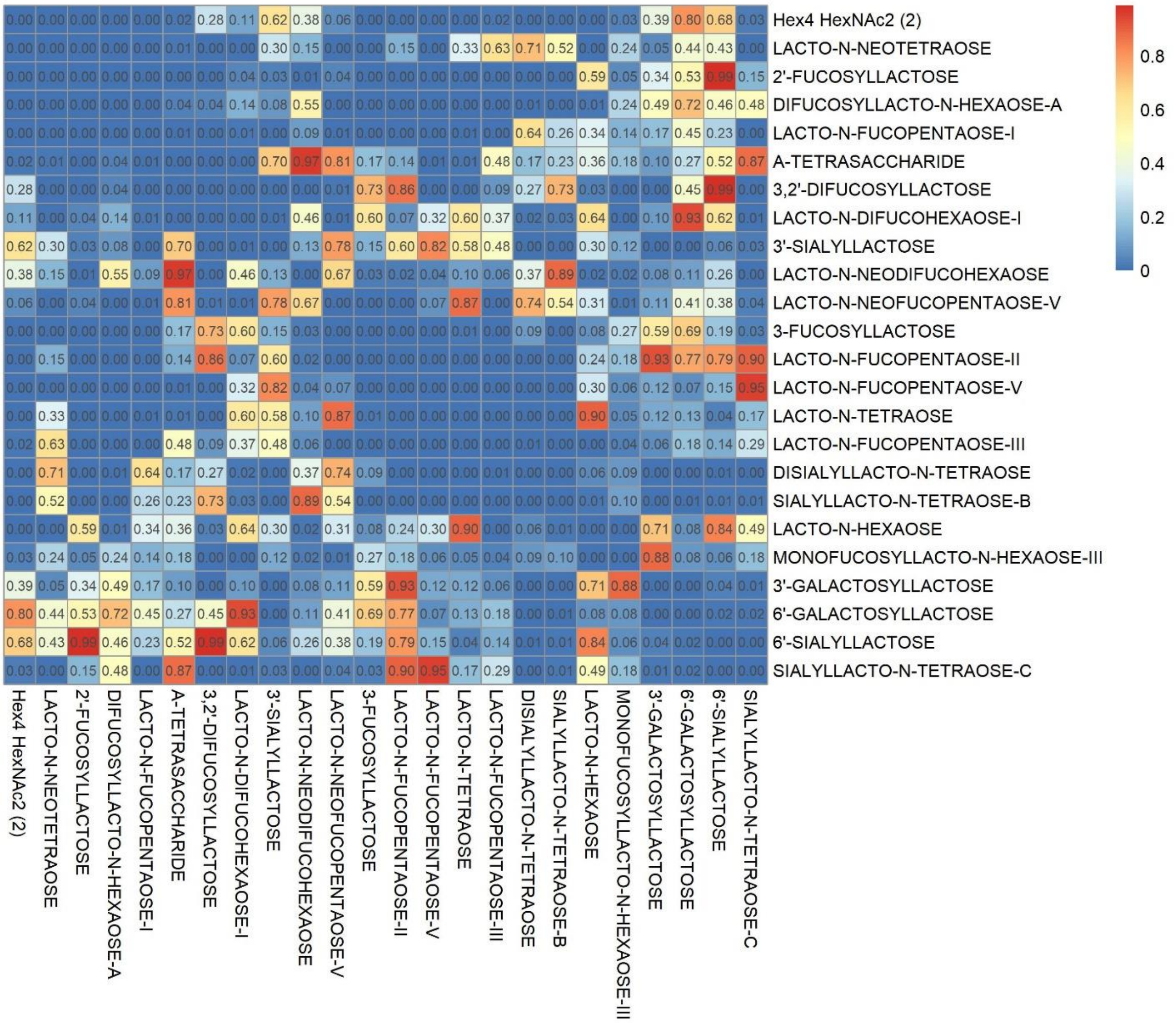

Supplementary Figure 2. Top: Pairwise correlations betweeh HMOs at visit V1. Bottom:corresponding p-values
