# Supplementary material for: Human milk oligosaccharide composition and associations with growth: results from an observational study in the US
Source: Front Nutr. 2023 Oct 3;10:1239349. doi: 10.3389/fnut.2023.1239349 (PMC10580431; doi:10.3389/fnut.2023.1239349)
Supplement: Supplementary file 6 [file Image_3.pdf]

Correlations between HMOs at 3 months

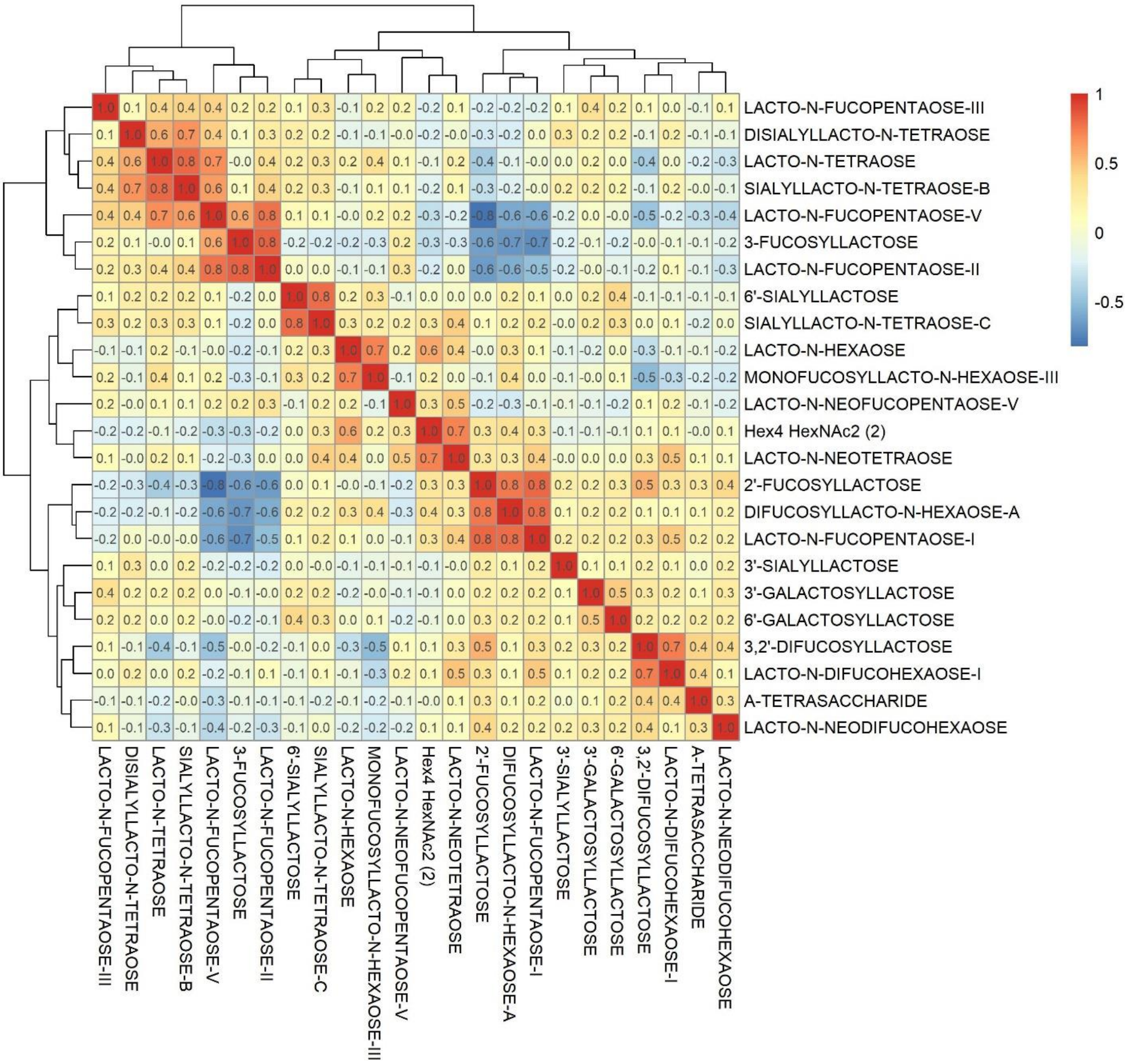

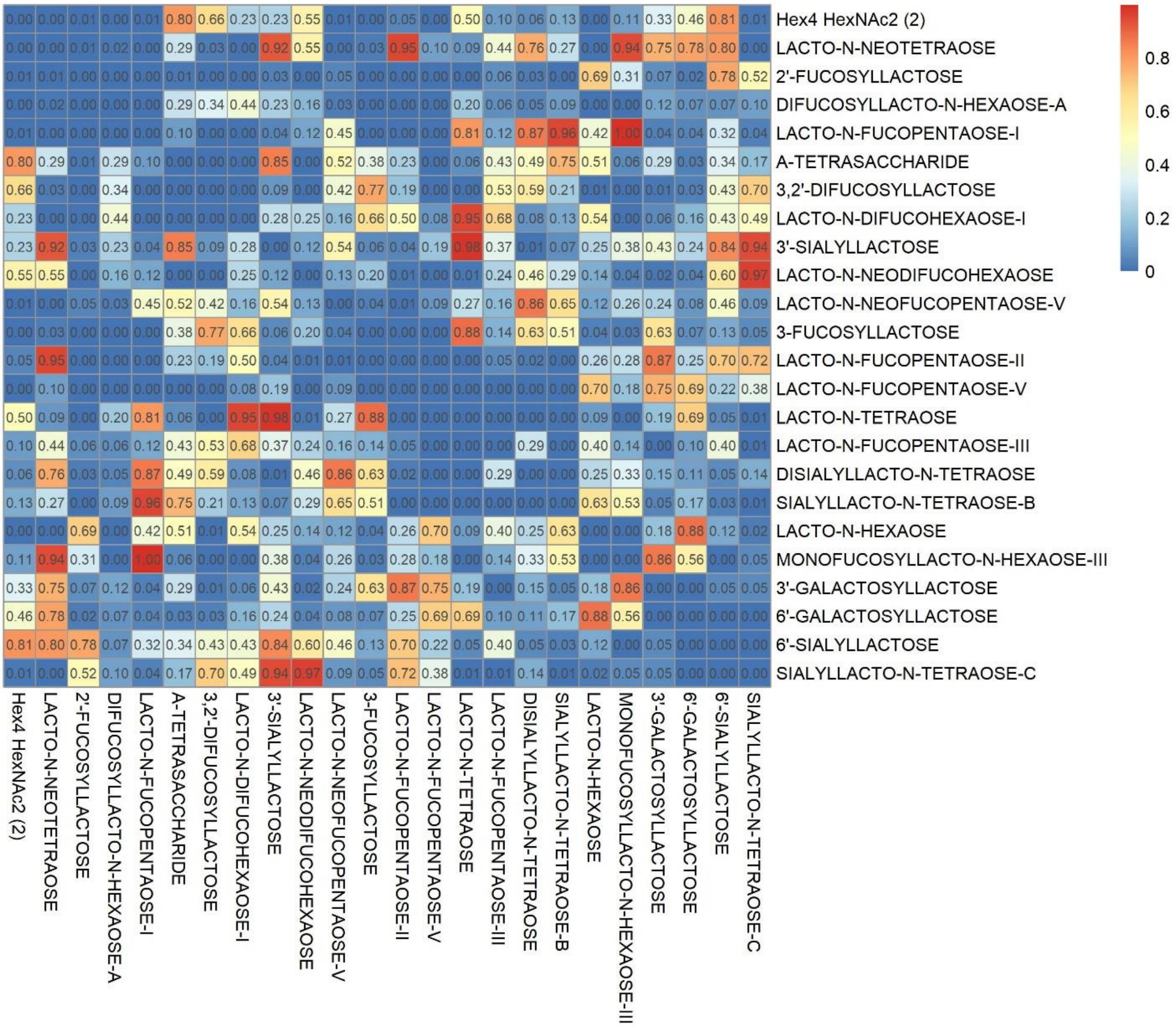

Supplementary Figure 3. Top: Pairwise correlations betweeh HMOs at visit V2. Bottom:corresponding p-values
