# Supplementary material for: Human milk oligosaccharide composition and associations with growth: results from an observational study in the US
Source: Front Nutr. 2023 Oct 3;10:1239349. doi: 10.3389/fnut.2023.1239349 (PMC10580431; doi:10.3389/fnut.2023.1239349)
Supplement: Supplementary file 7 [file Image_4.pdf]

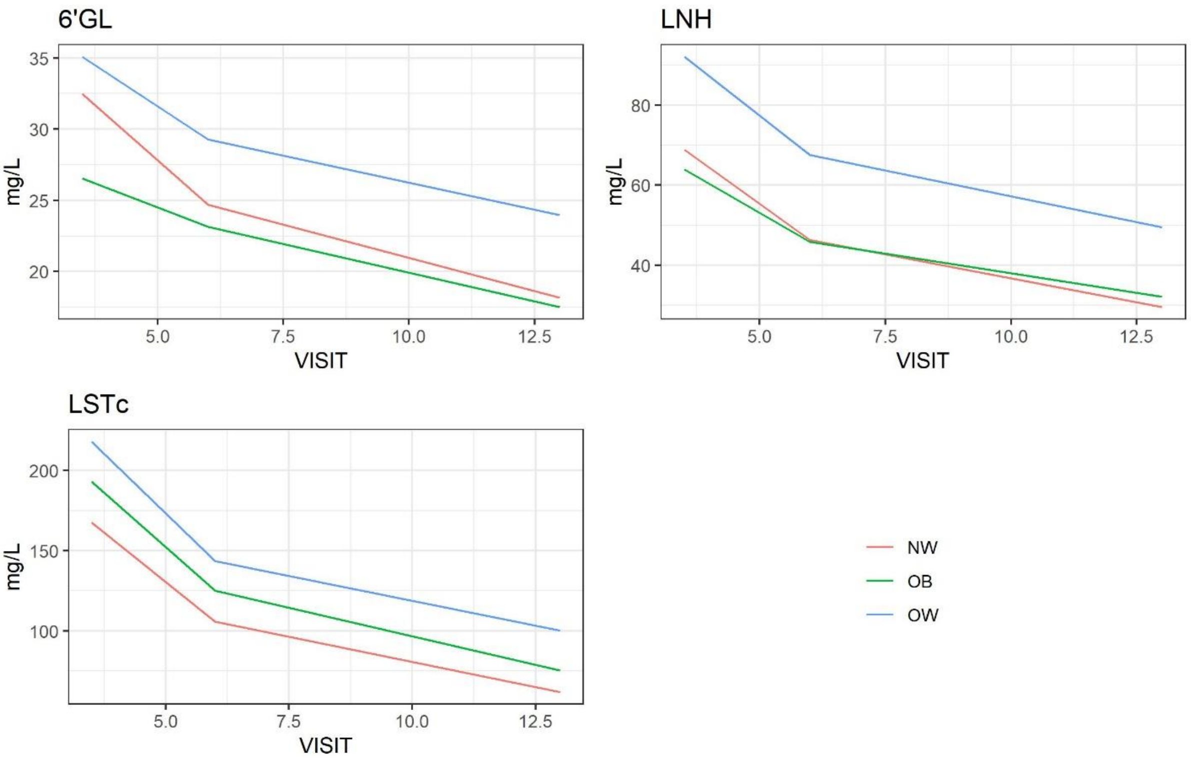

Supplementary Figure 4. Mean trajectories of these HMOs are significantly different. NW=Normal Weight, OB= Obese, OW = Overweight. The mean comparison was done with a functional ANOVA test (R package fdANOVA). On the x-axis, the time is measured in weeks
