# Supplementary material for: Human milk oligosaccharide composition and associations with growth: results from an observational study in the US
Source: Front Nutr. 2023 Oct 3;10:1239349. doi: 10.3389/fnut.2023.1239349 (PMC10580431; doi:10.3389/fnut.2023.1239349)
Supplement: Supplementary file 9 [file Image_6.pdf]

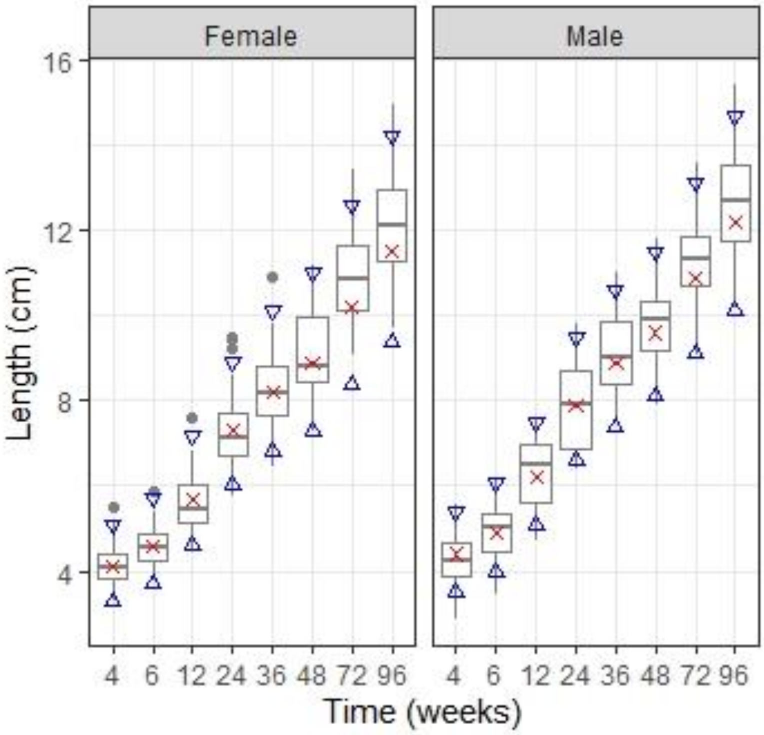

Supplementary Figure 6. Boxplots represent the weight of the children in the cohort, red crosses correspond to the median weight-for-age and blue triangles indicate the 5th and 95th percentiles of Length-for-age from the WHO
