# Supplementary material for: Human milk oligosaccharide composition and associations with growth: results from an observational study in the US
Source: Front Nutr. 2023 Oct 3;10:1239349. doi: 10.3389/fnut.2023.1239349 (PMC10580431; doi:10.3389/fnut.2023.1239349)
Supplement: Supplementary file 11 [file Image_8.pdf]

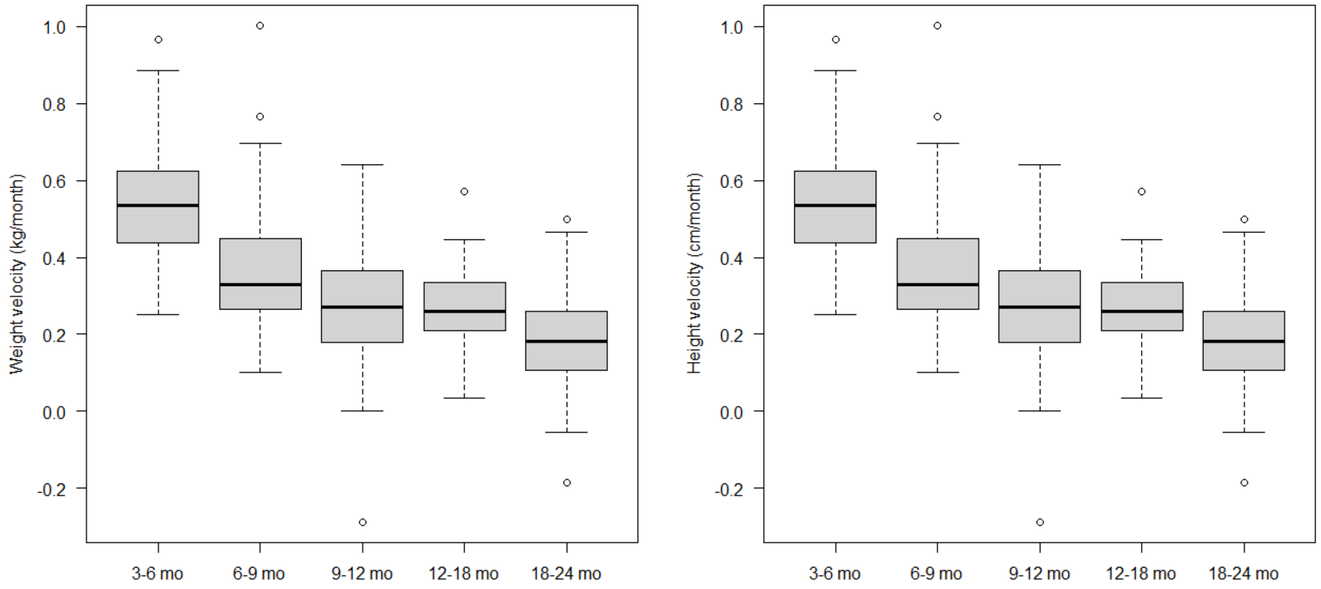

Supplementary Figure 8. Weight velocity is calculated as (weight(T1)-weight(T2))/(T2-T1). A similar formula was applied for height velocity.
