# Supplementary material for: Human milk oligosaccharide composition and associations with growth: results from an observational study in the US
Source: Front Nutr. 2023 Oct 3;10:1239349. doi: 10.3389/fnut.2023.1239349 (PMC10580431; doi:10.3389/fnut.2023.1239349)
Supplement: Supplementary file 12 [file Image_9.pdf]

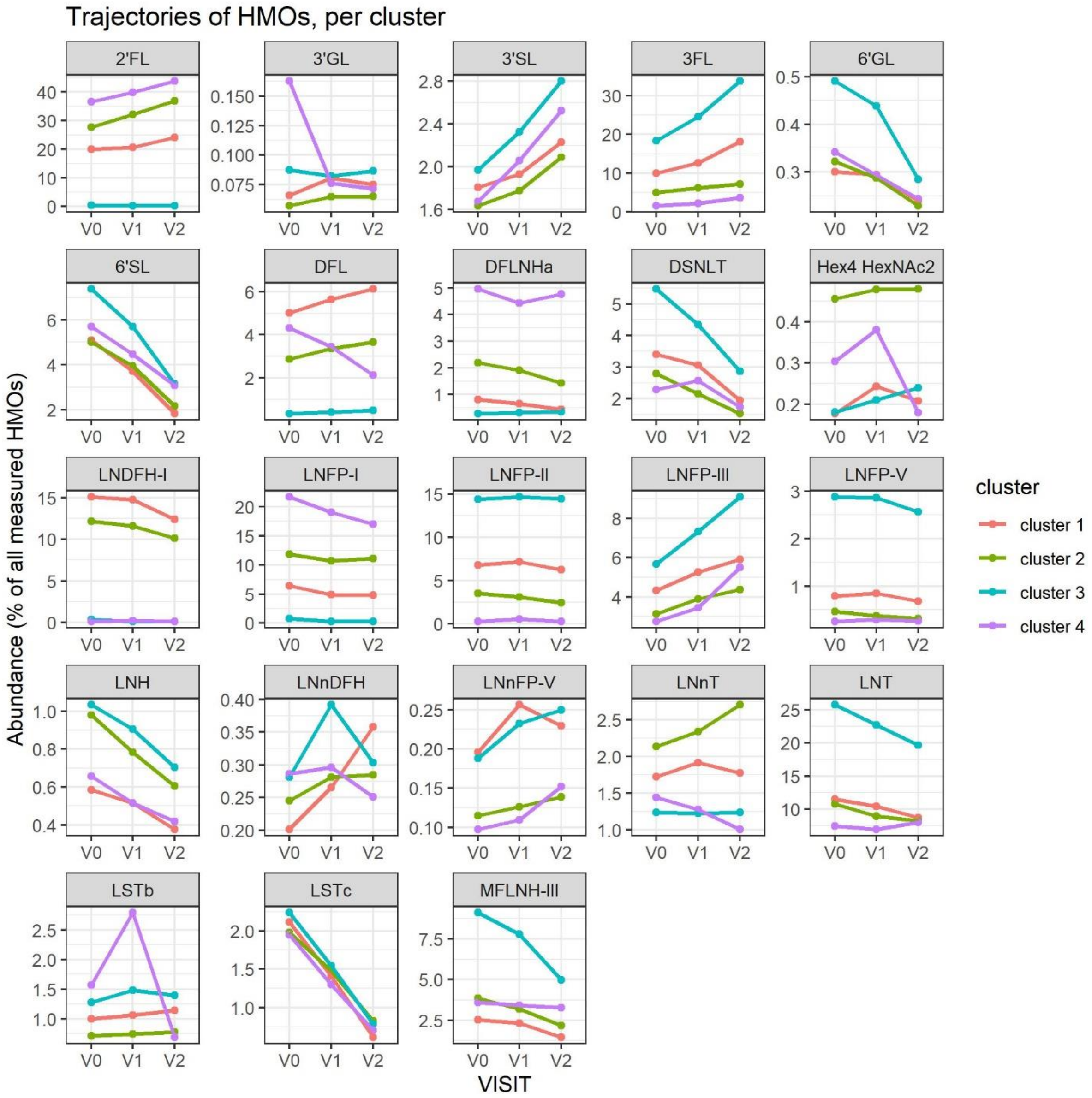

Supplementary Figure 9. Profiles of HMO clusters: each dot represents the average proportion of a HMO with respect to the total measured HMOs, in each cluster and for each time point. Several clustering variables show consistent temporal patterns: for example, 2'FL is consistently highest in cluster 4, while 3FL is consistently highest in cluster 3 (non-secretors), HEX is highest in cluster 2. Other HMOs (eg LSTc) do not show a strong difference between the clusters. V0=2-5 weeks, V1 = 6 weeks, V2=3 months.
